# Multilayer* 3D* Chirality and Its Synthetic Assembly

**DOI:** 10.34133/2019/6717104

**Published:** 2019-06-27

**Authors:** Guanzhao Wu, Yangxue Liu, Zhen Yang, Nandakumar Katakam, Hossein Rouh, Sultan Ahmed, Daniel Unruh, Kazimierz Surowiec, Guigen Li

**Affiliations:** ^1^Institute of Chemistry and BioMedical Sciences, School of Chemistry and Chemical Engineering, Nanjing University, Nanjing 210093, China; ^2^Department of Chemistry and Biochemistry, Texas Tech University, Lubbock, TX 79409-1061, USA

## Abstract

*3D* chirality of sandwich type of organic molecules has been discovered. The key element of this chirality is characterized by three layers of structures that are arranged nearly in parallel fashion with one on top and one down from the center plane. Individual enantiomers of these molecules have been fully characterized by spectroscopies with their enantiomeric purity measured by chiral HPLC. The absolute configuration was unambiguously assigned by X-ray diffraction analysis. This is the first multilayer* 3D* chirality reported and is anticipated to lead to a new research area of asymmetric synthesis and catalysis and to have a broad impact on chemical, medicinal, and material sciences in future.

## 1. Introduction

**Figure 1:**
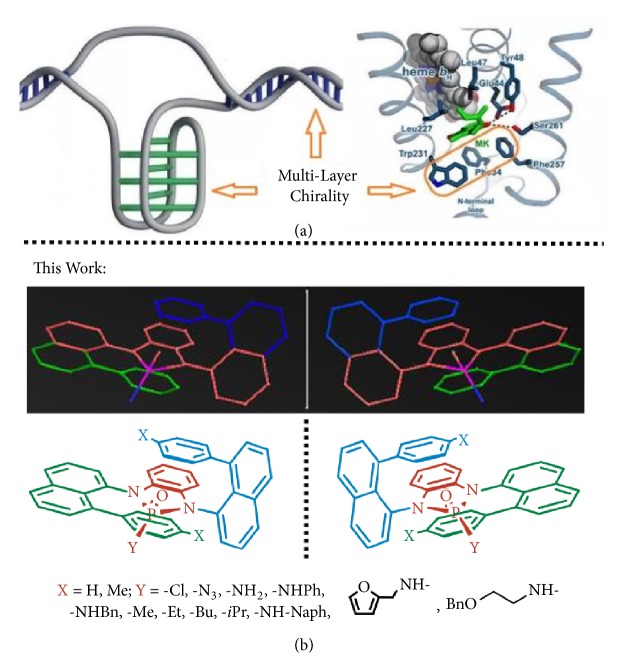
*Planar chirality.* (a) DNA i-motif and mycobacterial proteins. (b) Mirror models and structures of multilayer 3D molecules.

The origin of life is about chirality because of its existence in biomolecules, such as nucleic acids, proteins, and carbohydrates and its involving in biological mechanism in human beings, animals, and plants on the earth [1–6]. Chirality has also become increasingly important in pharmaceutical science and industry on the design, discovery, and development of drugs for enhancing their potency and selectivity [5, 6]. In this regard, asymmetric and catalytic synthesis of chiral compounds have been actively pursued for more than half a century to meet the needs of chemical and biomedical research [7–20]. Among the numerous chiral biomolecules and their complexes, DNA has attracted special attention in the chemistry community because it shows multilayer paired chirality along with their double- or single-strand and* i*-Motif backbones (Figure 1(a)) [21–23]. Proteins also often show multilayer chirality in their folding structures [24].

Even though multilayer chirality has existed in nature from the beginning of the living world, a strategy to mimic this functionality by using planar chirality has not been established yet to the best of our knowledge. So far, planar chirality has only been focused on two-layer arrangement which has been applied to a series of asymmetric reactions [25–34]. For example, Lautens and coworkers established a bulky and electron rich Qphos ligand for Pd-catalyzed cycloisomerization (carbohalogenation) reaction for the formation of versatile heterocycles in which the carbon iodine bond was regenerated at the end of the catalytic cycle [27–30]. Scheidt's group deployed planar-chiral NHC copper complexes for highly selective control of the delivery of the boron nucleophile to in situ formed imines to give medicinally relevant *α*-amidoboronates [31]. It is interesting that, during this work, the synthesis of both *α*-tosyl benzamide and enantioenriched potassium *α*-amido trifluoroborates would be regarded as GAP (Group-Assisted Purification) since their special functional groups enabled their purification to be performed simply by washing. Very recently, Krische and coworkers launched chromatographically stable chiral Iridium-PhanePhos catalysts and successfully utilized them for various important reactions including allene-fluoral reductive coupling, C-C coupling of methanol with dienes, and CF_3_-allenes [32, 33]. In the meanwhile, the two-layer chirality of imidazoline-based biaryl* P*,*N*-ligands has been elegantly designed for asymmetric alkyne conjugate addition and alkynylation reactions with excellent asymmetric induction [34].

In recent years, several other groups have made great progress on planar chiral and achiral Lewis acids of Ti, Zr, and Dy which showed effectiveness on polymerization processes and for achieving molecular magnetic hysteresis [35–38]. In addition, chiral two-layer helicenes have attracted much attention in material sciences [39, 40].

In all of above known cases, for Wilkinson's ferrocene, a transition metal is needed to anchor two planes for designing two-layered chiral catalysts. For optically pure [2,2] phanephos ligands, two covalent bonds are necessitated to assemble their two-layer chirality so as to efficiently control catalytic activity and enantioselectivity of asymmetric reactions. For noncovalent imidazoline-based biaryl* P*,*N*-ligands, the aromatic interaction between the fully fluorinated electron-deficient phenyl ring and the electron-efficient naphthyl ring plays a crucial role in stabilizing chiral planar stacking.

In this work, we present the discovery of an unprecedented chirality of multilayer and three dimensions. This chirality belongs to a type of Multilayer Organic Framework (*M*-LOF) of single organic molecules with* pseudo C*_*2*_-chiral characteristics. The key element of this chirality is characterized by three layers of planes that are arranged nearly in parallel fashion with one on top and one below the planar center as presented in Figure 1(b) (molecular models & chemical structures).

**Figure 2:**
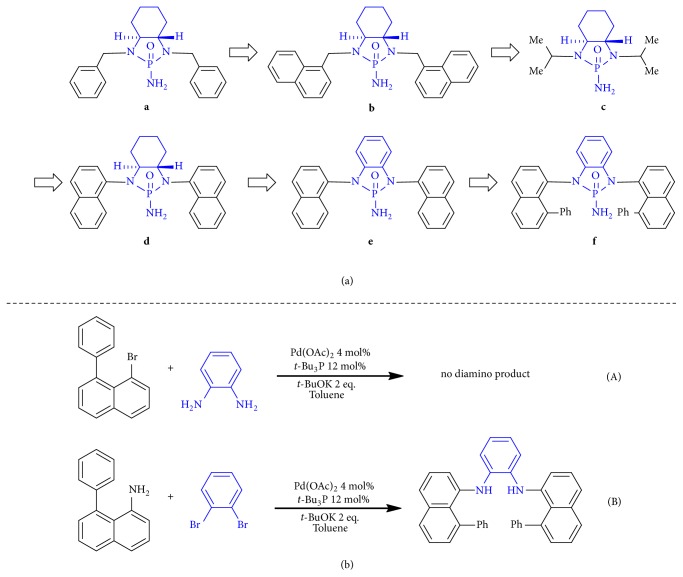
*Design and synthesis.* (a) Searching for more efficient GAP groups. (b) Synthesis of* N,N*-Diaryl benzene-1,2-diamines.

This discovery was resulted from our Group-Assisted Purification (GAP) chemistry by taking advantage of* N*-phosphonyl imine reagents and their usage for asymmetric reactions (Figure 2(a),** a-c**) [41–47]. In this project, we found that chiral and achiral* N*-phosphonyl- or* N*-phosphinyl groups avoided the formation of oily and sticky products; instead, they afforded special crystalline solids that can be purified simply by washing crude products with common petroleum solvents without the use of column chromatography and recrystallization. We also found these GAP groups can increase chemical yields [46], particularly for peptide synthesis, which is defined as Group-Assisted Synthesis (GASyn) [47]. GASyn chemistry has resulted in nearly quantitative yield for each step of polypeptide synthesis. In addition, GAP and GASyn chemistry has made it possible for the Fmoc-based peptide synthesis to be performed in solution phase for the first time [47]. In pharmaceutical industry, the syntheses of 1 kg of peptide and oligonucleotide drugs (10-15 mer products) and 3000-15000 kg of wastes are generated mostly from purification [48]. The GAP chemistry thus provides a unique environmentally friendly tool by taking advantages of both solution-phase and solid-phase syntheses [49–51] without many of their shortcomings. Indeed, GAP chemistry is the only chemical concept that combines the four aspects into one: reagents, reaction, separation, and purification; it requires the consideration of both reactants and products on their chemical and physical factors in regard to reactivity, selectivity, stability, and solubility. For asymmetric synthesis and catalysis, diastereo-, enantio-, and chemoselectivity have to be taken into account concurrently.

Typical GAP functionality is represented by* N,N*-phosphonyl amides with their development process described in Figure 2(a). Continuing the search for more efficient GAP functionality has been a main goal in our labs so that more environmentally friendly and greener syntheses, particularly, asymmetric synthesis, can be achieved. One of our strategies for this purpose is to replace* N,N*-dialkyl motifs of GAP functionality with their naphthyl counterparts, which has been proven to be promising to improve our GAP synthesis (Figure 2(a)-**d**). Furthermore, we would like to explore a new chirality pertaining to the chiral centers on the vicinal cyclohexyl diamine framework being removed, from which two individual enantiomers of* N,N*-phosphonyl GAP amides would be generated.

## 2. Results

At beginning, the synthesis of naphthyl-based GAP functionality was started with the Buchwald-Hartwig cross coupling [51, 52] of 1-bromo-8-phenylnaphthalene with (*1S,2S*)-cyclohexane-1,2-diamine or vicinal benzene diamine under a slightly modified condition consisting of Pd(OAc)_2_ and butylphosphine as catalysts and potassium* tert*-butoxide as a base additive. The reactions proceeded smoothly to give the corresponding coupling products in over 80% chemical yields (Figure 2(a)-**d** &** e**). The following steps consisting of cyclization of resulting* N,N*-protected diamines with phosphoryl trichloride in pyridine, displacement with sodium azide in DMF, and Pd/C-catalyzed hydrogenation were carried out to give* N,N*-phosphoryl amides over 65% yields overall. While product of Figure 2(a)-**d** showed the arrangement of di-1-nathphyl rings in* anti*-configuration; however, products of Figure 2(a)-**e** were formed as a mixture of* anti* and* syn* isomers, which can be separated via column chromatography. Increasing temperature of this mixture in dichloromethane to 50°C for over 5 hours, these two isomers reached equilibrium with two paired of enantiomeric peaks as 25% for each as revealed by chiral HPLC analysis.

We next turned our attention to changing* N,N*-phosphonyl GAP functionality by introducing a phenyl group onto 1-bromonaphthalene at position 8 to restrict the axial flexibility so as to control* anti*-isomerism of* N,N*-di-1-nathphyls predominantly. As usual, the synthesis was started with the Buchwald-Hartwig cross coupling [52, 53] between vicinal benzene diamine and 1-bromo-8-phenylnaphthalene. Surprisingly, the coupling reaction resulted in a complex mixture with nearly no formation of the* N*,*N*-protected diamino product under the same condition as the above (Figure 2(b), equation (A)). Other similar modifications on catalytic conditions all failed to result in any promising outcomes. The strategy was thus changed to use 1,2-dibromobenzene for the reaction with 1-amino-8-phenylnaphthalene under Pd(OAc)_2_-based catalytic systems. Among three common phosphines (Bu_3_P,* t-*Bu_3_P, and Ph_3_P), only* t-*Bu_3_P gave promising results when reaction was performed by refluxing in toluene for three days. The use of Pd(OAc)_2_ and* t-*Bu_3_P in 4 mol% and 10 mol%, respectively, afforded the coupling product,* N,N*-bis(8-phenylnaphthalen-1-yl)benzene-1,2-diamine in a chemical yield of 14%. (Figure 2(b), equation (B)). Although the yield of this step is still much lower than that of normal cross coupling reactions, it enabled the following steps to be continued to achieve* N,N*-phosphonyl GAP amides (Figure 4).

**Figure 3:**
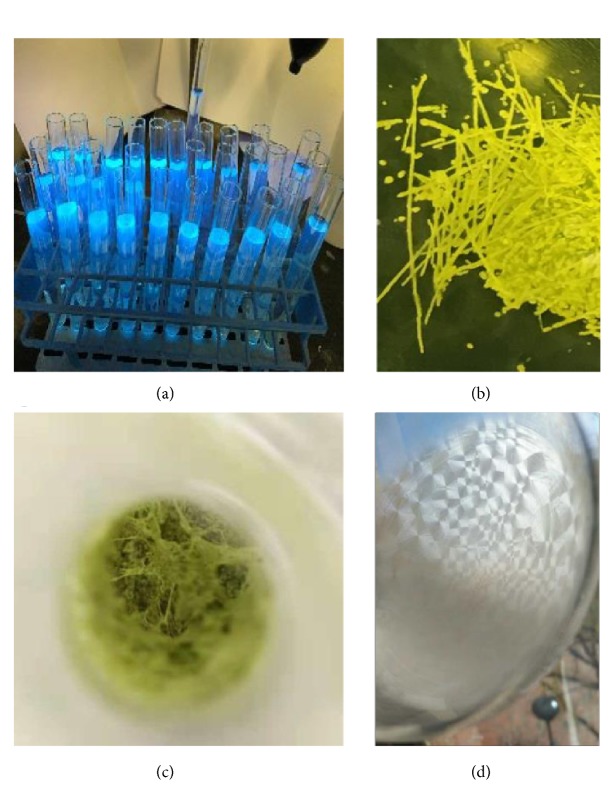
*Physical showing of diamino products.* (a), (b), and (c) Physical appearance of* N,N*-bis(8-phenylnaphthalen-1-yl)benzene-1,2-diamine. (d) Hornet' nest pattern of product** 21**.

While performing the C-N coupling reactions,* N,N*-bis(8-phenylnaphthalen-1-yl)benzene-1,2-diamine (**9**) was found to shine a green color under UV irradiation (Figure 3(a)). A major unknown side product also shone with purple color under UV irradiation. This product was formed as various long strings of firm solids with up to a length of a few centimeters (Figure 3(b)); when they were evaporated in a relatively smaller flask, a nest-web of firm solid was formed, which filled the majority of the flask space (Figure 3(c)). Single crystals of this product were obtained from a cosolvent of EtOAc/hexane during a period of two weeks. The X-ray structural analysis revealed that each phenyl ring is nearly parallel to the naphthylene ring of their neighboring 8-phenylnaphthalen-1-yl group (see SI). There is no hydrogen bond formation that exists between the two amino groups, and there is no intermolecular hydrogen bonding observed either. Interestingly, the intermolecular packing of* N,N*-bis(8-phenylnaphthalen-1-yl)benzene-1,2-diamine showed a helix type of structural arrangement (see SI).

**Figure 4:**
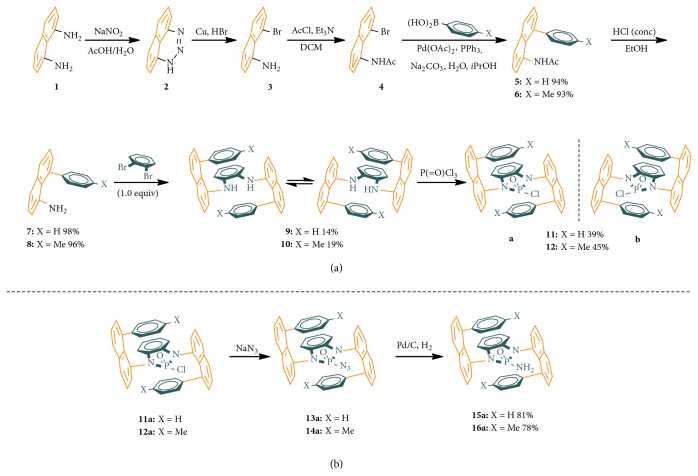
*Synthetic routes.* (a) Synthesis of 8-phenylnaphthalen-1-amine precursors and* N,N*-phosphonyl chlorides. (b) Synthesis of* N,N*-phosphonyl azides and amides.

The total synthesis (Figure 4) of this new chirality was started by oxidative cyclization by reacting naphthalene-1,8-diamine with sodium nitrite in aqueous media containing acetic acid to give 1H-naphtho[1,8-de][1,2, 3]triazine (**2**) [54–59]. Ring-opening of this triazine was performed by treating it with metal copper in hydrogen bromide. The resulting 8-bromonaphthalen-1-amine (**3**) was converted to* N*-(8-bromonaphthalen-1-yl)acetamide (**4**), which was followed by Suzuki coupling to afford* N*-acetyl 8-phenylnaphthalen-1-amine (**5**). 8-Phenylnaphthalen-1-amine (**7**) was generated by acidic hydrolysis with concentrated aqueous HCl, which was subjected to the Buchwald-Hartwig C-N double cross couplings.

The* N,N*-diamino cross coupling product is anticipated to exist in the form of two major enantiomeric conformers (**9**), which are not distinguishable by chiral HPLC. However, after they are cyclized into 2-chloro-1,3-bis(8-phenylnaphthalen-1-yl)-1,3-dihydrobenzo[d][1,3, 2]diazaphosphole 2-oxide (**11**), two individual enantiomers can be analyzed and separated through analytical and preparative chiral HPLC columns, respectively. At this cyclization step, previous conditions for forming amides by treating diamines with in triethyl amines or pyridine failed to give any cyclization product for case Figure 2(a)-**f**, although they worked well for cases Figure 2(a)-**a** to Figure 2(a)-**e**. The cyclization reaction was successfully conducted by deprotonating* N,N*-bis(8-phenylnaphthalen-1-yl)benzene-1,2-diamines (**9**) with* n*-butyl lithium followed by treating with trichlorophosphine oxide at -78°C in dried THF. The cyclization difficulty is probably due to the large steric effect from the phenyl group on position 8 of the naphthalene ring which would also be responsible for the incomplete conversion of this reaction. A yield of 39% was obtained at this step while the remaining 1,2-diamine starting material was recovered for reuse* via* column chromatography in 48% yield.

Attempts were made to obtain single crystals of individual enantiomers of 2-chloro-1,3-bis(8-phenylnaphthalen-1-yl)-1,3-dihydrobenzo[d][1,3, 2]diazaphosphole 2-oxide (*N*-phosphonyl chloride,** 11a**) without success. Pleasantly, we achieved X-ray diffraction analysis of crystals of 2-amino-1,3-bis(8-phenylnaphthalen-1-yl)-1,3-dihydrobenzo[d][1,3, 2]diazaphosphole 2-oxide (**15a**) (see SI), which was derived from the above* N,N*-phosphonyl chloride through the formation of the corresponding azide precursor that eventually enabled the absolute structure of the* N*-phosphonyl chloride to be determined indirectly. Also, the effort of obtaining single crystals for 2-azido-1,3-bis(8-phenylnaphthalen-1-yl)-1,3-dihydrobenzo[d][1,3, 2]diazaphosphole 2-oxide (**13a**) was unsuccessful. We also failed to find chiral analytical HPLC conditions for the azide racemic samples after many efforts were made. Quantitative yields were obtained at the azide displacement step when the reaction was performed in acetonitrile at 90°C. The resulting* N,N*-phosphonyl azide was directly subjected to the final Pd/C-catalyzed hydrogenation which showed quantitative yields as well.

**Figure 5:**
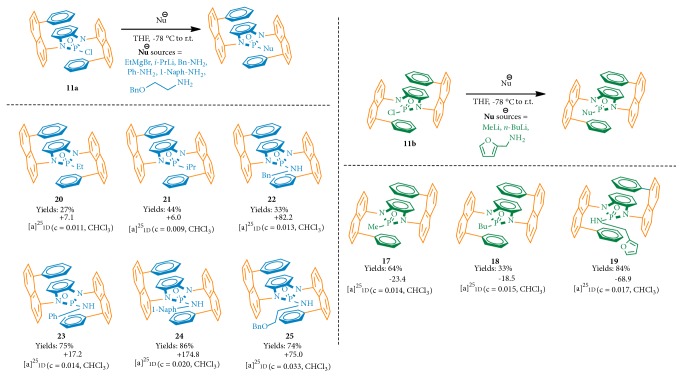
Substitution reactions of enantiomers of* N,N*-phosphonyl chloride.

After individual enantiomers of* N,N*-phosphonyl chlorides were generated through preparative chiral HPLC, we investigated their synthetic potentials by transferring them into various building blocks. At first, methyl lithium was subjected to the nucleophilic substitution reaction with an isomer (**11b**) of* N,N*-phosphonyl chloride (Figure 5). The reaction was finished after running in dried THF at -78°C for 30 min and then was raised to room temperature for 6 h. A yield of 64% was achieved without observation of any racemization; this was confirmed by running the same reaction using racemic* N,N*-phosphonyl chloride with the resulting product subjected to chiral HPLC analysis (see SI). As we expected the bulky sandwich center plane can prevent the axial rotation which is necessary for racemization. Two other nucleophiles,* n*-butyl lithium and furan-2-ylmethanamine, were also employed for the reaction with the enantiomer under similar conditions to give 2-butyl-1,3-bis(8-phenylnaphthalen-1-yl)-1,3-dihydrobenzo[d][1,3, 2]diazaphosphole 2-oxide (**18**) and 2-((furan-2-ylmethyl)amino)-1,3-bis(8-phenylnaphthalen-1-yl)-1,3-dihydrobenzo[d][1,3, 2]diazaphosphole 2-oxide (**19**) in yields of 33% and 84%, respectively (Figure 5).

Based on the availability of enantiomerically pure* N,N*-phosphonyl chlorides generated from preparative HPLC, we then examined other nucleophiles for the similar reaction with the other isomer (**11a**). These nucleophiles include ethyl magnesium bromide,* i*-propyl lithium, phenylmethanamine, aniline, naphthalen-1-amine, and 2-(benzyloxy)ethan-1-amine. All of these nucleophiles reacted with the* N,N*-phosphonyl chloride isomer (**11a**) smoothly to give good to high yields as shown in Figure 5. It should be noted that, for amino nucleophiles, their treatment with* n*-butyl lithium was performed for the preformation of lithium alkylamide or lithium arylamide prior to the nucleophilic substitution reaction. All of these products (Figure 5,** 20-25**) were proven to be stable at room temperature since decomposition was not detected for more than one month without any inner gas protection.

Interestingly, while conducting* i*-propyl lithium-based substitution, we found that the resulting product of 2-isopropyl-1,3-bis(8-phenylnaphthalen-1-yl)-1,3-dihydrobenzo[d][1,3, 2]diazaphosphole 2-oxide (**21**) was formed in the shown by a hornet' nest pattern of white color after evaporation was operated to dryness (Figure 3(d)). Most hornet' nest units consist of an almost identical shape of various sizes. Other alkyl derivatives did not show this phenomenon, indicating* i-*propyl group would be useful for controlling the properties of certain materials for future research.

**Figure 6:**
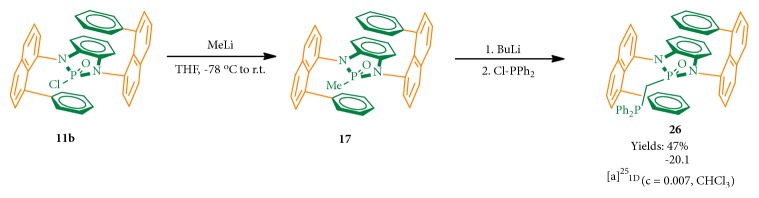
Conversion of one isomer of* N,N*-phosphonyl chloride to phosphine.

**Figure 7:**
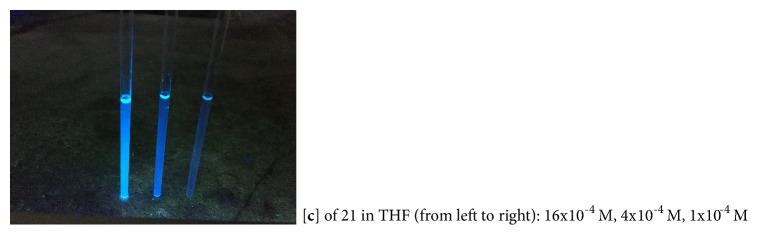
AIE-type showing of multilayer* 3D* chirality.

To further investigate the synthetic potential of this novel chirality, we converted an enantiomerically pure isomer (**11b**) of* N,N*-phosphonyl chloride into a chiral phosphine ligand which belongs to the most active field in asymmetric catalysis (Figure 6,** 26**) [59–64]. The key step was to conduct the deprotonation of 2-methyl-1,3-bis(8-phenylnaphthalen-1-yl)-1,3-dihydrobenzo[d][1,3, 2]diazaphosphole 2-oxide by using* n*-BuLi followed by a nucleophilic substitution reaction with chlorodiphenylphosphine. This two-step synthesis gave an excellent overall yield of 47%. Since this new ligand is anchored by GAP functionality, it is anticipated to be recyclable for reuse* via* simple GAP purification [64]. It should be pointed out that chiral sandwich molecules including this ligand showed high stability at room temperature without observing racemization as confirmed by chiral HPLC analysis (see SI). The work on improving yields of the* N,N*-di-coupling reaction and applications of this new chirality are currently being investigated in our labs. Also, since multilayer* 3D* molecules in this work exhibit not only fluorescence but also AIE activity as represented by product** 21** in Figure 7 [65, 66], interdisciplinary collaboration on this project with material science community will be conducted in the near future.

## 3. Discussion

In conclusion, we have discovered a novel organic sandwich chirality showing multilayer three dimensions. The absolute structure has been unambiguously confirmed by X-ray diffraction analysis of signal crystals. The key element of this chirality is characterized by three planes that are arranged nearly in parallel fashion with one on top and one below the centre layer. The resulting 3D-multilayer chiral products have been converted into various building blocks, particularly, anchored by an* N,N*-phosphonyl GAP group to give new chiral phosphine ligands for asymmetric chemistry. The GAP-catalyst strategy resulted in the design, synthesis and application of more environmentally friendly materials and catalysts for recycling and reuse. The work would be anticipated to have a great impact on chemical, pharmaceutical, and material sciences in the future. The investigation of fully carbon-carbon bond-anchored multilayer* 3D* chirality of sandwich types is currently being conducted in our labs.

## 4. Materials and Methods

Unless otherwise stated, all reactions were magnetically stirred and conducted in oven-dried glassware in anhydrous solvents under Ar, applying standard Schlenk techniques. Solvents and liquid reagents as well as solutions of solid or liquid reagents were added via syringes, stainless steel, or polyethylene cannulas through rubber septa or through a weak Ar counterflow. Cooling baths were prepared in Dewar vessels, filled with ice/water (0°C) or dry ice/acetone (-78°C). Heated oil baths were used for reactions requiring elevated temperatures. Solvents were removed under reduced pressure at 40-65°C using a rotavapor. All given yields are isolated yields of chromatographically and NMR spectroscopically materials.

All commercially available chemicals were used as received without further purification. Solvents were obtained as follows: MeOH, EtOH,* i*PrOH, Hexane, EA, ether, DCM, THF, CH_3_CN, and toluene are delivered from an Innovation Technology solvent system.

The ^1^H and ^13^C NMR spectra were recorded in CDCl_3_ or DMSO-*d*_6_ on 400 MHz and 500 MHz instruments with TMS as internal standard. For referencing of the ^1^H NMR spectra, the residual solvent signal (*δ* = 7.26 for CDCl_3_ and *δ* = 2.50 for DMSO-*d*_6_) was used. In the case of the ^13^C NMR spectra, the signal of solvents (*δ* = 7.16 for CDCl_3_ and *δ* = 39.52 for DMSO-*d*_6_) were used. Chemical shifts (*δ*) were reported in ppm with respect to TMS. Data are represented as follows: chemical shift, multiplicity (s = singlet, d = doublet, t = triplet, and m = multiplet), coupling constant (*J*, Hz), and integration. ^31^P NMR spectra were referenced to external H_3_PO_4_ (0.00 ppm). HRMS analyses were carried out using a TOF-MS instrument with an ESI source. For HPLC analysis of enantioselectivity and separation, experiments were performed on Thermo Fisher Scientific UltiMate 3000 HPLC using Daicel chiral columns. Data were collected by Chromeleon program. All solvents (*i*PrOH, Hexane, EA, DCM) used were HPLC-grade solvents without further purification. Optical rotations were measured with a Rudolph Research Analytical APIV/2W Polarimeter at the indicated temperature with a sodium lamp. Measurements were performed in a 2 mL with concentrations (g/ (10 mL)) reported in the corresponding solvent. X-ray crystallographic analysis was performed with a SMART CCD and a P4 diffractometer.

X-ray data were collected on a Bruker PLATFORM three circle diffractometer equipped with an APEX II CCD detector and operated at 1350 W (40kV, 30 mA) to generate (graphite monochromated) Mo K*α* radiation (*λ* = 0.71073 Å). Crystals were transferred from the vial and placed on a glass slide in polyisobutylene. A Zeiss Stemi 305 microscope was used to identify a suitable specimen for X-ray diffraction from a representative sample of the material. The crystal and a small amount of the oil were collected on a MῑTiGen cryoloop and transferred to the instrument where it was placed under a cold nitrogen stream (Oxford) maintained at 100K throughout the duration of the experiment. The samples were optically centered with the aid of a video camera to insure that no translations were observed as the crystal was rotated through all positions. A unit cell collection was then carried out. After it was determined that the unit cell was not present in the CCDC database a sphere of data was collected. Omega scans were carried out with a 120 sec/frame exposure time and a rotation of 2.0° per frame. After data collection, the crystal was measured for size, morphology, and color. These values are reported in Tables [Supplementary-material supplementary-material-1] & [Supplementary-material supplementary-material-1]. After data collection, the unit cell was redetermined using a subset of the full data collection. Intensity data were corrected for Lorentz, polarization, and background effects using the Bruker program APEX 3. A semiempirical correction for adsorption was applied using the program* SADABS* [67, 68]. The* SHELXL-2014 *[68, 69], series of programs, was used for the solution and refinement of the crystal structure. Hydrogen atoms bound to carbon and nitrogen atoms were located in the difference Fourier map and were geometrically constrained using the appropriate AFIX commands. Due to poor crystal diffraction quality of the crystals the rigid-bond restraint RIGU was applied globally during the final refinements.

## Data Availability

All data are available in the manuscript or supplementary materials.

## Supplementary Materials

Supplementary Materials
* Figure S1*: HPLC spectrum of racemic** 11**.* Figure S2*: HPLC spectrum of isomer** 11a**.* Figure S3*: HPLC spectrum of isomer** 11b**.* Figure S4*: HPLC spectrum of racemic** 15**.* Figure S5*: HPLC spectrum of isomer** 15a**.* Figure S6*: HPLC spectrum of isomer** 15b**.* Figure S7*: HPLC spectrum of racemic** 16**.* Figure S8*: HPLC spectrum of isomer** 16a**.* Figure S9*: HPLC spectrum of isomer** 16b**.* Figure S10*: HPLC spectrum of racemic** 17**.* Figure S11*: HPLC spectrum of isomer** 17**.* Figure S12*: X-ray structure of** 9**.* Figure S13*: crystal used for single crystal X-ray diffraction analysis.* Figure S14*: X-ray structure of** 15a**.* Figure S15*: crystal used for single crystal X-ray diffraction analysis.* Table S1*: crystal data and structure refinement for** 9**.* Table S2*: atomic coordinates ( x 10^4^) and equivalent isotropic displacement parameters (Å^2^ x 10^3^) for** 9**. U(eq) is defined as one-third of the trace of the orthogonalized Uij tensor.* Table S3*: bond lengths [Å] and angles [°] for** 9**.* Table S4*: anisotropic displacement parameters (Å^2^ x 10^3^) for** 9**. The anisotropic displacement factor exponent takes the form: -2 *π*^2^ [ h^2^ a*∗*^2^U^11^+… + 2 h k a*∗* b*∗* U^12^].* Table S5*: hydrogen coordinates (x 10^4^) and isotropic displacement parameters (Å^2^ x 10^3^) for** 9**.* Table S6*: crystal data and structure refinement for** 15a**.* Table S7*: atomic coordinates ( x 10^4^) and equivalent isotropic displacement parameters (Å^2^ x 10^3^) for** 15a**. U(eq) is defined as one-third of the trace of the orthogonalized Uij tensor.* Table S8*: bond lengths [Å] and angles [°] for** 15a**.* Table S9*: anisotropic displacement parameters (Å^2^ x 10^3^) for** 15a**. The anisotropic displacement factor exponent takes the form: -2 *π*^2^ [ h^2^ a*∗*^2^U^11^+… + 2 h k a*∗* b*∗* U^12^].* Table S10*: hydrogen coordinates (x 10^4^) and isotropic displacement parameters (Å^2^ x 10^3^) for** 15a**.* Table S11*: hydrogen bonds for** 15a** where hydrogen bonds with H..A < r(A) + 2.000 Å and <DHA > 110° are listed.
