# Supplementary material for: Multilayer 3D Chirality and Its Synthetic Assembly
Source: Research (Wash D C). 2019 Jun 27;2019:6717104. doi: 10.34133/2019/6717104 (PMC6750085; doi:10.34133/2019/6717104)
Supplement: Supplementary Materials — Figure S1: HPLC spectrum of racemic 11. Figure S2: HPLC spectrum of isomer 11a. Figure S3: HPLC spectrum of isomer 11b. Figure S4: HPLC spectrum of racemic 15. Figure S5: HPLC spectrum of isomer 15a. Figure S6: HPLC spectrum of isomer 15b. Figure S7: HPLC spectrum of racemic 16. Figure S8: HPLC spectrum of isomer 16a. Figure S9: HPLC spectrum of isomer 16b. Figure S10: HPLC spectrum of racemic 17. Figure S11: HPLC spectrum of isomer 17. Figure S12: X-ray structure of 9. Figure S13: crystal used for single crystal X-ray diffraction analysis. Figure S14: X-ray structure of 15a. Figure S15: crystal used for single crystal X-ray diffraction analysis. Table S1: crystal data and structure refinement for 9. Table S2: atomic coordinates ( x 104) and equivalent isotropic displacement parameters (Å2 x 103) for 9. U(eq) is defined as one-third of the trace of the orthogonalized Uij tensor. Table S3: bond lengths [Å] and angles [°] for 9. Table S4: anisotropic displacement parameters (Å2 x 103) for 9. The anisotropic displacement factor exponent takes the form: -2 π2 [ h2 a∗2U11+… + 2 h k a∗ b∗ U12]. Table S5: hydrogen coordinates (x 104) and isotropic displacement parameters (Å2 x 103) for 9. Table S6: crystal data and structure refinement for 15a. Table S7: atomic coordinates ( x 104) and equivalent isotropic displacement parameters (Å2 x 103) for 15a. U(eq) is defined as one-third of the trace of the orthogonalized Uij tensor. Table S8: bond lengths [Å] and angles [°] for 15a. Table S9: anisotropic displacement parameters (Å2 x 103) for 15a. The anisotropic displacement factor exponent takes the form: -2 π2 [ h2 a∗2U11+… + 2 h k a∗ b∗ U12]. Table S10: hydrogen coordinates (x 104) and isotropic displacement parameters (Å2 x 103) for 15a. Table S11: hydrogen bonds for 15a where hydrogen bonds with H..A < r(A) + 2.000 Å and 110° are listed. [file 6717104.f1.zip › 6717104.f1/6717104_SupplDesc.docx]

**Figure S1**. HPLC spectrum of racemic **11**. **Figure S2**. HPLC spectrum of isomer **11a**. **Figure S3**. HPLC spectrum of isomer **11b**. **Figure S4**. HPLC spectrum of racemic **15**. **Figure S5**. HPLC spectrum of isomer **15a**. **Figure S6**. HPLC spectrum of isomer **15b**. **Figure S7**. HPLC spectrum of racemic **16**. **Figure S8**. HPLC spectrum of isomer **16a**. **Figure S9**. HPLC spectrum of isomer **16b**. **Figure S10**. HPLC spectrum of racemic **17**. **Figure S11**. HPLC spectrum of isomer **17**. **Figure S12.** X-ray structure of **9**. **Figure S13.** Crystal used for single crystal X-ray diffraction analysis. **Figure S14.** X-ray structure of **15a**. **Figure S15.** Crystal used for single crystal X-ray diffraction analysis. **Table S1.** Crystal data and structure refinement for **9**. **Table S2.** Atomic coordinates ( x 10^4^) and equivalent isotropic displacement parameters (Å^2^ x 10^3^) for **9**. U(eq) is defined as one third of the trace of the orthogonalized Uij tensor. **Table S3.** Bond lengths [Å] and angles [°] for **9**. **Table S4.** Anisotropic displacement parameters (Å^2^ x 10^3^) for **9**. The anisotropic displacement factor exponent takes the form: -2 π^2^ [ h^2^ a*^2^U^11^ + ... + 2 h k a* b* U^12^]. **Table S5.** Hydrogen coordinates (x 10^4^) and isotropic displacement parameters (Å^2^ x 10^3^) for **9**. **Table S6.**  Crystal data and structure refinement for **15a**. **Table S7.**  Atomic coordinates ( x 10^4^) and equivalent isotropic displacement parameters (Å^2^ x 10^3^) for **15a**. U(eq) is defined as one third of the trace of the orthogonalized Uij tensor. **Table S8.** Bond lengths [Å] and angles [°] for **15a**. **Table S9.** Anisotropic displacement parameters (Å^2^ x 10^3^) for **15a**. The anisotropic displacement factor exponent takes the form: -2 π^2^ [ h^2^ a*^2^U^11^ + ... + 2 h k a* b* U^12^ ]. **Table S10.** Hydrogen coordinates (x 10^4^) and isotropic displacement parameters (Å^2^ x 10^3^) for **15a**. **Table S11.** Hydrogen bonds for **15a** where hydrogen bonds with H..A < r(A) + 2.000 Å and <DHA > 110° are listed.
