# Supplementary material for: Multilayer 3D Chirality and Its Synthetic Assembly
Source: Research (Wash D C). 2019 Jun 27;2019:6717104. doi: 10.34133/2019/6717104 (PMC6750085; doi:10.34133/2019/6717104)
Supplement: Supplementary Materials — Figure S1: HPLC spectrum of racemic 11. Figure S2: HPLC spectrum of isomer 11a. Figure S3: HPLC spectrum of isomer 11b. Figure S4: HPLC spectrum of racemic 15. Figure S5: HPLC spectrum of isomer 15a. Figure S6: HPLC spectrum of isomer 15b. Figure S7: HPLC spectrum of racemic 16. Figure S8: HPLC spectrum of isomer 16a. Figure S9: HPLC spectrum of isomer 16b. Figure S10: HPLC spectrum of racemic 17. Figure S11: HPLC spectrum of isomer 17. Figure S12: X-ray structure of 9. Figure S13: crystal used for single crystal X-ray diffraction analysis. Figure S14: X-ray structure of 15a. Figure S15: crystal used for single crystal X-ray diffraction analysis. Table S1: crystal data and structure refinement for 9. Table S2: atomic coordinates ( x 104) and equivalent isotropic displacement parameters (Å2 x 103) for 9. U(eq) is defined as one-third of the trace of the orthogonalized Uij tensor. Table S3: bond lengths [Å] and angles [°] for 9. Table S4: anisotropic displacement parameters (Å2 x 103) for 9. The anisotropic displacement factor exponent takes the form: -2 π2 [ h2 a∗2U11+… + 2 h k a∗ b∗ U12]. Table S5: hydrogen coordinates (x 104) and isotropic displacement parameters (Å2 x 103) for 9. Table S6: crystal data and structure refinement for 15a. Table S7: atomic coordinates ( x 104) and equivalent isotropic displacement parameters (Å2 x 103) for 15a. U(eq) is defined as one-third of the trace of the orthogonalized Uij tensor. Table S8: bond lengths [Å] and angles [°] for 15a. Table S9: anisotropic displacement parameters (Å2 x 103) for 15a. The anisotropic displacement factor exponent takes the form: -2 π2 [ h2 a∗2U11+… + 2 h k a∗ b∗ U12]. Table S10: hydrogen coordinates (x 104) and isotropic displacement parameters (Å2 x 103) for 15a. Table S11: hydrogen bonds for 15a where hydrogen bonds with H..A < r(A) + 2.000 Å and 110° are listed. [file 6717104.f1.zip › 6717104.f1/supple figures.doc]

Supplementary Materials for

**Multi-Layer *3D* Chirality and its Synthetic Assembly**

Guanzhao Wu,^1,2,†^ Yangxue Liu,^2,†^ Zhen Yang,^1,†^ Nandakumar Katakam,^2^ Hossein Rouh,^2^ Sultan Ahmed,^2^ Daniel Unruh,^2^ Kazimierz Surowiec,^2^ and Guigen Li^1,2,*^

*^1^ Institute of Chemistry and BioMedical Sciences, School of Chemistry and Chemical Engineering, Nanjing University, Nanjing, 210093, China.*

*^2^ Department of Chemistry and Biochemistry, Texas Tech University, Lubbock, Texas 79409-1061, USA.*

**^*^**Correspondence should be addressed to Guigen Li; [guigen.li@ttu.edu](mailto:guigen.li@ttu.edu)

**^†^**These authors contributed equally to this work.

1 Synthetic Procedures
  1.1 Synthesis and Characterization of **9** & **10**.

A round bottom flask, *t*-Bu_3_P (492.51 μmol, 0.996 g, 10% in hexane) and dibromide (1.64 mmol, 0.387 g) was added slowly into a degassed solution of *t*-BuOK (6.57 mmol, 0.737 g) and Pd(OAc)_2_ (164.17 μmol, 0.033 g) in toluene, following by the degassed toluene solution of amine (4.1 mmol, 0.900 g), flashed with argon. The reaction mixture was heated to 110 °C and stirred for 72 h. The solvent was evaporated under reduced pressure and the residue was purified by flash chromatography on silica gel (hexane/DCM, 10:1 to 4:1) to afford pure product.

**N1,N2-bis(8-phenylnaphthalen-1-yl)benzene-1,2-diamine (9):** yellow-green solid, 230 mg, 14% yield; ^1^H NMR (400 MHz, CHLOROFORM-D) δ 7.87 (d, *J* = 8.2 Hz, 1H), 7.51 (d, *J* = 8.0 Hz, 1H), 7.44 (t, *J* = 7.6 Hz, 1H), 7.37 (t, *J* = 7.8 Hz, 1H), 7.23 (d, *J* = 7.7 Hz, 2H), 7.18 (d, *J* = 6.9 Hz, 1H), 7.03 (dd, *J* = 5.8, 3.6 Hz, 1H), 7.00 – 6.90 (m, 3H), 6.86 (t, *J* = 7.2 Hz, 1H), 6.74 (dd, *J* = 5.9, 3.5 Hz, 1H), 4.86 (s, 1H).

^13^C NMR (101 MHz, CHLOROFORM-D) δ = 142.76, 140.15, 137.98, 136.17, 134.84, 128.86, 128.79, 128.65, 127.92, 127.48, 126.21, 124.70, 123.57, 121.46, 121.05, 118.21, 114.32.

HRMS (ESI- TOF) *m*/*z* [C_38_H_28_N_2_ + H] ^+^ calcd for 513.2325, found 509.2000 (-4H).

**N1,N2-bis(8-(p-tolyl)naphthalen-1-yl)benzene-1,2-diamine (10):** Yellow-green solid, 233 mg, 19% yield; ^1^H NMR (400 MHz, CHLOROFORM-D) δ 7.87 – 7.79 (m, 2H), 7.46 (dd, *J* = 8.1, 1.1 Hz, 2H), 7.44 – 7.39 (m, 2H), 7.36 – 7.31 (m, 2H), 7.16 – 7.09 (m, 8H), 6.99 – 6.94 (m, 2H), 6.74 – 6.65 (m, 6H), 4.88 (s, 2H), 1.77 (d, *J* = 3.4 Hz, 6H).

^13^C NMR (101 MHz, CHLOROFORM-D) δ = 138.97, 138.49, 136.80, 136.29, 135.15, 133.31, 127.75, 127.53, 127.48, 124.98, 123.61, 122.31, 119.90, 119.65, 116.28, 112.67, 19.65.

HRMS (ESI- TOF) *m*/*z* [C_40_H_32_N_2_ + H] ^+^ calcd for 541.2638, found 537.2310 (-4H).

1.2 Synthesis and Characterization of **11** & **12**.

An oven-dried argon-flushed Schlenk flask was charged with dry THF solution of **6** (1.11 mmol, 0.570 g), at -78 °C, stirred for 10 min, a solution of *n*-BuLi (2.22 mmol, 0.142 g, 1.6 M in hexane) was added dropwise and stirred for 0.5 h. POCl_3_(2.78 mmol, 0.426 g) was added dropwise via syringe into the mixture at -78 °C, the resultant mixture was allowed to warm to r.t. and stirred for overnight. The solvent was evaporated in vacuo for flash chromatography column directly, eluted by mixed solvents of hexane/ethyl acetate (50:1 to 10:1) to afford pure product.

**2-chloro-1,3-bis(8-phenylnaphthalen-1-yl)-1,3-dihydrobenzo[d][1,3,2]diazaphosphole 2-oxide (11b):** pale yellow solid, 257 mg, 39% yield; [α]_D_^25^ = -212.7 (*c* = 0.002, CHCl_3_).

^1^H NMR (400 MHz, CHLOROFORM-D) δ 7.96 – 7.91 (m, 2H), 7.84 (ddd, *J* = 13.6, 8.2, 1.2 Hz, 2H), 7.63 (d, *J* = 7.7 Hz, 1H), 7.50 (td, *J* = 7.6, 2.0 Hz, 2H), 7.41 (ddd, *J* = 8.1, 7.1, 3.4 Hz, 2H), 7.34 – 7.28 (m, 2H), 7.22 (dt, *J* = 8.7, 3.4 Hz, 2H), 7.17 – 7.14 (m, 1H), 7.11 (ddd, *J* = 7.3, 2.1, 1.4 Hz, 2H), 7.02 (tt, *J* = 7.4, 1.2 Hz, 1H), 6.85 (tt, *J* = 7.5, 1.2 Hz, 1H), 6.77 (d, *J* = 6.3 Hz, 1H), 6.68 (tt, *J* = 7.7, 1.2 Hz, 1H), 6.64 – 6.56 (m, 3H), 6.31 – 6.23 (m, 2H), 5.99 – 5.92 (m, 1H).

^13^C NMR (101 MHz, CHLOROFORM-D) δ = 141.75, 141.71, 138.97, 138.36, 136.52, 136.28, 134.73, 134.54, 134.09, 133.90, 132.64, 132.19, 131.59, 131.29, 130.91, 130.75, 130.71, 130.44, 129.85, 129.82, 129.66, 129.61, 129.36, 129.31, 128.56, 126.81, 126.28, 126.12, 125.98, 125.79, 125.64, 125.26, 124.93, 120.54, 120.44, 111.40, 110.36.

^31^P NMR (162 MHz, CHLOROFORM-D) δ = 9.77.

HRMS (ESI- TOF) *m*/*z* [C_38_H_26_ClN_2_OP + H] ^+^ calcd for 593.1544, found 593.1555.

**2-chloro-1,3-bis(8-(p-tolyl)naphthalen-1-yl)-1,3-dihydrobenzo[d][1,3,2]diazaphosphole 2-oxide (12):** brown solid, 84 mg, 45% yield.

^1^H NMR (400 MHz, CHLOROFORM-D) δ 7.92 (t, *J* = 7.6 Hz, 2H), 7.81 (dd, *J* = 13.4, 8.2 Hz, 2H), 7.53 – 7.45 (m, 3H), 7.38 (ddd, *J* = 13.7, 8.5, 5.9 Hz, 3H), 7.22 (d, *J* = 7.0 Hz, 1H), 7.12 (dd, *J* = 15.9, 7.1 Hz, 4H), 6.92 (s, 1H), 6.62 (dt, *J* = 27.5, 7.3 Hz, 3H), 6.47 – 6.34 (m, 2H), 6.22 (d, *J* = 7.6 Hz, 1H), 6.02 (d, *J* = 7.3 Hz, 1H), 5.90 (d, *J* = 7.6 Hz, 1H), 2.18 (s, 3H), 1.99 (s, 3H).

^13^C NMR (101 MHz, CHLOROFORM-D) δ = 139.02, 138.83, 138.73, 138.48, 136.55, 136.31, 135.76, 135.49, 134.90, 134.71, 134.28, 134.08, 132.61, 132.12, 131.29, 131.25, 131.03, 130.90, 130.77, 130.45, 129.92, 129.84, 129.22, 129.11, 128.51, 127.62, 126.07, 125.82, 125.51, 125.46, 125.32, 120.51, 120.43, 111.10, 110.35, 21.18, 21.14.

^31^P NMR (162 MHz, CHLOROFORM-D) δ = 9.58.

HRMS (ESI- TOF) *m*/*z* [C_40_H_30_ClN_2_OP + H] ^+^ calcd for 621.1857, found 621.1855.

1.3 Synthesis and Characterization of **15a** & **16a**.

**11a or 12a** (168.62 μmol, 0.100 g) and NaN_3_ (505.85 μmol, 0.033 g) was added to an argon flashed round bottom flask, add dried CH_3_CN as solvent with syringe, stir reaction for 4 h at 90 °C. Quenched reaction with H_2_O and extracted with ethyl acetate, dried for next step directly. Pd/C (33.72 μmol, 0.036 g, 10%) are added to the sealed flask with product from last step, after three vacuum/H_2_ cycles to replace the air inside with hydrogen, keep a balloon of hydrogen connected to the reaction system and transfer MeOH as solvent with syringe. Reaction mixture was stirred at r.t. for 12 h. Filter reaction mixture with celite pad. Filtrate was evaporated under reduced vacuo for chromatography column, eluted by mixed solvents of hexane/ethyl acetate (10:1 to 3:1) to give pure product.

**2-amino-1,3-bis(8-phenylnaphthalen-1-yl)-1,3-dihydrobenzo[d][1,3,2]diazaphosphole 2-oxide (15a)**: pale white solid, 87 mg; 81% yield; [α]_D_^25^ = +104.4 (*c* = 0.012, CHCl_3_).

^1^H NMR (400 MHz, CHLOROFORM-D) δ 7.89 (ddd, *J* = 8.2, 5.4, 2.8 Hz, 3H), 7.81 (dd, *J* = 8.3, 1.3 Hz, 1H), 7.69 (d, *J* = 7.2 Hz, 1H), 7.57 – 7.52 (m, 1H), 7.47 – 7.43 (m, 2H), 7.40 (t, *J* = 7.6 Hz, 2H), 7.29 (t, *J* = 7.6 Hz, 1H), 7.20 (dd, *J* = 5.5, 1.0 Hz, 2H), 7.07 (ddd, *J* = 6.4, 5.6, 1.6 Hz, 2H), 6.87 – 6.82 (m, 2H), 6.70 (d, *J* = 7.7 Hz, 1H), 6.66 – 6.57 (m, 3H), 6.56 – 6.50 (m, 1H), 6.33 (t, *J* = 7.5 Hz, 1H), 6.14 (d, *J* = 7.6 Hz, 1H), 5.70 – 5.64 (m, 1H), 2.08 (d, *J* = 5.7 Hz, 2H).

^13^C NMR (101 MHz, CHLOROFORM-D) δ = 143.52, 141.55, 139.65, 137.64, 136.49, 136.04, 134.99, 134.83, 134.66, 134.48, 132.23, 131.97, 131.92, 131.80, 131.21, 130.98, 130.62, 130.12, 129.99, 129.80, 129.55, 129.18, 128.89, 128.42, 126.64, 126.26, 125.70, 125.54, 125.51, 124.96, 119.34, 119.21, 109.86, 109.70.

^31^P NMR (162 MHz, CHLOROFORM-D) δ = 8.16.

HRMS (ESI- TOF) *m*/*z* [C_38_H_28_N_3_OP + H] ^+^ calcd for 574.2043, found 602.1855 (+CO).

**2-amino-1,3-bis(8-(p-tolyl)naphthalen-1-yl)-1,3-dihydrobenzo[d][1,3,2]diazaphosphole 2-oxide (16a)**: pale white solid, 22 mg, 78% yield; [α]_D_^25^ = -100.9 (*c* = 0.002, MeOH).

^1^H NMR (400 MHz, CHLOROFORM-D) δ 7.97 – 7.92 (m, 3H), 7.86 (dd, *J* = 8.2, 1.2 Hz, 1H), 7.61 (t, *J* = 7.7 Hz, 2H), 7.54 (dd, *J* = 5.9, 1.4 Hz, 1H), 7.50 (d, *J* = 7.4 Hz, 1H), 7.45 (ddd, *J* = 9.2, 6.0, 2.0 Hz, 2H), 7.27 (dd, *J* = 7.1, 1.3 Hz, 1H), 7.13 (dd, *J* = 7.1, 1.3 Hz, 2H), 7.08 (d, *J* = 7.4 Hz, 1H), 7.02 – 6.97 (m, 1H), 6.92 – 6.87 (m, 1H), 6.64 (t, *J* = 7.5 Hz, 1H), 6.59 – 6.47 (m, 4H), 6.17 (s, 1H), 6.12 (d, *J* = 7.6 Hz, 1H), 5.67 (d, *J* = 7.7 Hz, 1H), 2.36 (d, *J* = 5.9 Hz, 3H), 2.20 – 2.15 (m, 2H), 2.08 (s, 3H). ^13^C NMR (101 MHz, CHLOROFORM-D) δ = 140.63, 139.66, 138.50, 137.63, 136.52, 136.37, 136.05, 135.16, 135.13, 134.91, 134.73, 131.85, 131.74, 131.37, 130.58, 130.29, 130.22, 130.09, 129.80, 129.46, 128.95, 128.38, 127.38, 126.87, 126.53, 126.51, 125.54, 125.32, 125.30, 125.00, 119.28, 119.16, 119.12, 109.88, 109.28, 29.79, 21.10.

^31^P NMR (162 MHz, CHLOROFORM-D) δ = 8.12.

HRMS (ESI- TOF) *m*/*z* [C_40_H_32_N_3_OP + H] ^+^ calcd for 602.2356, found 602.2351.

1.4 Synthesis and Characterization of **17**, **18**, **20** & **21**.

A dried argon-flushed Schlenk flask was charged with dry THF solution of **11a/11b** (0.1 mmol), at -78 °C, a solution of CH_3_Li (0.12 mmol, 1.6 M in hexane), C_2_H_5_MgBr (0.12 mmol, 3 M in diethyl ether), (CH_3_)_2_CHLi (0.12 mmol, 0.7 M in pentane) or *n*-BuLi (0.12 mmol, 1.6 M in hexane) was added dropwise. The resultant mixture was stirred at -78 °C for 0.5 h, allowed to warm to r.t. and stirred for 6 h. Monitor by TLC plate and dry for column directly (Hexane / Ethyl acetate = 50:1 to 10:1).

**2-methyl-1,3-bis(8-phenylnaphthalen-1-yl)-1,3-dihydrobenzo[d][1,3,2]diazaphosphole 2-oxide (17):** pale yellow solid, 23 mg, 64% yield; [α]_D_^25^ = -23.4 (*c* = 0.014, CHCl_3_).

^1^H NMR (400 MHz, DMSO-D6) δ 8.16 (dd, *J* = 11.5, 8.3 Hz, 3H), 8.08 (d, *J* = 8.2 Hz, 1H), 7.72 (td, *J* = 7.8, 5.4 Hz, 2H), 7.67 – 7.57 (m, 3H), 7.40 – 7.26 (m, 6H), 7.21 (d, *J* = 7.5 Hz, 1H), 7.05 – 6.96 (m, 3H), 6.83 – 6.67 (m, 4H), 6.50 (d, *J* = 7.5 Hz, 2H), 6.01 (d, *J* = 7.6 Hz, 1H), 0.72 (d, *J* = 16.2 Hz, 3H).

^13^C NMR (101 MHz, CHLOROFORM-D) δ = 142.05, 140.83, 138.72, 136.80, 136.10, 136.00, 135.57, 135.10, 135.04, 134.97, 131.75, 131.42, 131.35, 131.17, 130.91, 130.19, 129.86, 129.16, 128.84, 128.81, 128.76, 128.36, 127.78, 127.06, 125.44, 125.30, 124.74, 124.67, 124.40, 123.86, 118.49, 118.37, 109.88, 109.13, 13.11.

^31^P NMR (162 MHz, DMSO-D6) δ = 29.09.

HRMS (ESI- TOF) *m*/*z* [C_39_H_29_N_2_OP + H] ^+^ calcd for 573.2090, found 573.2086.

**2-ethyl-1,3-bis(8-phenylnaphthalen-1-yl)-1,3-dihydrobenzo[d][1,3,2]diazaphosphole 2-oxide (20):** pale yellow solid, 9 mg, 27% yield; [α]_D_^25^ = +7.1 (*c* = 0.011, CHCl_3_).

^1^H NMR (400 MHz, DMSO-D6) δ 8.16 (t, *J* = 9.0 Hz, 3H), 8.07 (d, *J* = 8.3 Hz, 1H), 7.71 (tt, *J* = 23.2, 11.5 Hz, 3H), 7.65 – 7.58 (m, 2H), 7.44 – 7.28 (m, 5H), 7.26 (t, *J* = 6.1 Hz, 2H), 6.95 (dd, *J* = 16.4, 7.6 Hz, 3H), 6.77 (t, *J* = 7.5 Hz, 2H), 6.70 (dd, *J* = 13.5, 6.3 Hz, 2H), 6.49 (t, *J* = 13.8 Hz, 2H), 5.90 (d, *J* = 7.8 Hz, 1H), 0.91 (dtd, *J* = 30.5, 15.2, 7.3 Hz, 3H), 0.19 (dt, *J* = 19.8, 7.5 Hz, 3H).

^13^C NMR (126 MHz, dmso) δ = 142.15, 141.41, 139.32, 137.47, 136.45, 136.38, 136.10, 135.71, 135.10, 135.00, 132.82, 132.77, 132.59, 132.04, 131.59, 131.10, 130.32, 129.73, 129.64, 129.56, 129.46, 129.42, 129.38, 129.23, 128.66, 128.02, 127.10, 126.42, 126.15, 125.70, 125.57, 125.49, 125.23, 119.34, 119.30, 110.46, 109.95, 22.34, 6.85.

^31^P NMR (162 MHz, DMSO-D6) δ = 31.30.

HRMS (ESI- TOF) *m*/*z* [C_40_H_31_N_2_OP + H] ^+^ calcd for 587.2247, found 587.2238.

**2-isopropyl-1,3-bis(8-phenylnaphthalen-1-yl)-1,3-dihydrobenzo[d][1,3,2]diazaphosphole 2-oxide (21):** pale yellow solid, 16 mg, 44% yield; [α]_D_^25^ = +6.0 (*c* = 0.009, CHCl_3_).

^1^H NMR (400 MHz, DMSO-D6) δ 8.08 (dd, *J* = 11.1, 4.1 Hz, 3H), 7.98 (dd, *J* = 8.3, 1.1 Hz, 1H), 7.75 – 7.58 (m, 3H), 7.54 (ddd, *J* = 8.3, 7.1, 1.4 Hz, 2H), 7.37 (d, *J* = 7.3 Hz, 1H), 7.27 (dt, *J* = 4.8, 2.9 Hz, 4H), 7.13 (dd, *J* = 7.0, 1.3 Hz, 1H), 7.08 (d, *J* = 4.5 Hz, 1H), 6.99 (d, *J* = 7.3 Hz, 1H), 6.83 (tt, *J* = 7.5, 1.2 Hz, 1H), 6.79 – 6.63 (m, 4H), 6.59 (t, *J* = 7.7 Hz, 1H), 6.51 (dd, *J* = 7.7, 0.9 Hz, 1H), 6.31 (s, 1H), 5.65 (d, *J* = 7.7 Hz, 1H), 1.19 – 1.08 (m, 1H), 0.42 (dd, *J* = 18.2, 6.8 Hz, 3H), -0.27 (dd, *J* = 18.6, 6.7 Hz, 3H).

^13^C NMR (101 MHz, DMSO-D6) δ = 142.63, 141.80, 140.06, 138.05, 136.77, 136.69, 136.66, 136.10, 135.36, 135.25, 134.17, 134.12, 134.06, 134.03, 132.65, 131.49, 130.96, 130.84, 130.46, 130.43, 130.14, 130.10, 129.94, 129.67, 129.61, 128.67, 128.28, 127.23, 126.75, 126.67, 126.62, 126.20, 125.96, 125.72, 119.81, 119.67, 111.00, 110.44, 28.94, 17.92, 16.43.

^31^P NMR (162 MHz, DMSO-D6) δ = 33.18.

HRMS (ESI- TOF) *m*/*z* [C_41_H_33_N_2_OP + H] ^+^ calcd for 601.2403, found 601.2397.

**2-butyl-1,3-bis(8-phenylnaphthalen-1-yl)-1,3-dihydrobenzo[d][1,3,2]diazaphosphole 2-oxide (18):** pink solid, 14 mg, 33% yield; [α]_D_^25^ = -18.5 (*c* = 0.015, CHCl_3_).

^1^H NMR (400 MHz, DMSO-D6) δ 8.15 (dd, *J* = 13.4, 5.0 Hz, 3H), 8.07 (dd, *J* = 8.3, 0.9 Hz, 1H), 7.72 (t, *J* = 7.8 Hz, 3H), 7.62 (dd, *J* = 8.0, 7.2 Hz, 2H), 7.41 – 7.30 (m, 5H), 7.26 (dd, *J* = 7.1, 1.2 Hz, 1H), 7.20 (s, 1H), 6.99 – 6.91 (m, 3H), 6.85 – 6.65 (m, 4H), 6.51 (t, *J* = 18.0 Hz, 2H), 5.90 (d, *J* = 7.7 Hz, 1H), 1.16 (ddd, *J* = 20.0, 13.7, 4.1 Hz, 2H), 0.95 – 0.87 (m, 2H), 0.48 (t, *J* = 7.2 Hz, 3H).

^13^C NMR (101 MHz, CHLOROFORM-D) δ = 141.99, 140.62, 138.85, 136.91, 135.94, 135.85, 135.49, 134.98, 134.79, 134.66, 132.28, 132.23, 131.61, 131.06, 131.03, 130.83, 130.23, 129.00, 128.78, 128.67, 128.58, 128.41, 128.08, 126.92, 125.79, 125.51, 125.12, 124.93, 124.72, 124.66, 124.38, 123.80, 118.33, 118.21, 109.67, 109.10, 28.23, 27.65, 23.52, 22.50.

^31^P NMR (162 MHz, DMSO-D6) δ = 30.01.

HRMS (ESI- TOF) *m*/*z* [C_42_H_35_N_2_OP + H] ^+^ calcd for 615.2560, found 615.2557.

1.5 Synthesis and Characterization of **19** & **22**-**25**.

A dried argon-flushed Schlenk flask was charged with dry THF solution of **different amines** (0.2 mmol), at -78 °C, a solution of *n*-BuLi (0.15 mmol, 1.6 M in hexane) was added dropwise and stirred for 0.5 h. **11a/11b** (0.1 mmol) was added dropwise via syringe into the mixture at -78 °C, the resultant mixture was allowed to warm to r.t. and stirred for 6 h. Monitor by TLC plate and dry for column directly (Hexane / Ethyl acetate = 50:1 to 10:1).

**2-(phenylamino)-1,3-bis(8-phenylnaphthalen-1-yl)-1,3-dihydrobenzo[d][1,3,2]diazaphosphole 2-oxide (23):** yellow solid, 24 mg, 75% yield; [α]_D_^25^ = +17.2 (*c* = 0.014, CHCl_3_).

^1^H NMR (400 MHz, DMSO-D6) δ 8.21 (d, *J* = 8.1 Hz, 1H), 8.16 (d, *J* = 8.2 Hz, 1H), 8.11 (d, *J* = 8.1 Hz, 1H), 8.05 (d, *J* = 8.3 Hz, 1H), 7.79 (t, *J* = 7.8 Hz, 1H), 7.62 (ddd, *J* = 12.9, 11.6, 6.3 Hz, 4H), 7.44 (dd, *J* = 15.6, 7.5 Hz, 3H), 7.35 (d, *J* = 14.0 Hz, 1H), 7.26 (d, *J* = 6.9 Hz, 1H), 7.22 (d, *J* = 7.0 Hz, 1H), 7.14 (s, 1H), 7.02 (d, *J* = 7.2 Hz, 1H), 6.96 (dd, *J* = 15.0, 7.3 Hz, 3H), 6.79 (dd, *J* = 13.5, 7.3 Hz, 4H), 6.76 – 6.69 (m, 2H), 6.55 (d, *J* = 7.8 Hz, 2H), 6.41 (d, *J* = 29.8 Hz, 1H), 6.34 (d, *J* = 7.6 Hz, 1H), 5.88 (d, *J* = 7.4 Hz, 1H).

^13^C NMR (126 MHz, dmso) δ = 142.54, 141.12, 139.62, 138.95, 136.97, 135.93, 135.66, 133.93, 133.80, 133.55, 133.41, 131.74, 131.37, 131.19, 131.17, 131.09, 131.06, 130.35, 130.09, 130.07, 129.97, 129.92, 129.43, 129.14, 129.11, 128.39, 128.37, 126.99, 126.79, 126.29, 126.07, 125.98, 125.55, 125.53, 125.34, 124.64, 124.60, 121.85, 119.69, 119.66, 119.61, 119.24, 109.87, 109.66.

^31^P NMR (162 MHz, DMSO-D6) δ = 3.14.

HRMS (ESI- TOF) *m*/*z* [C_44_H_32_N_3_OP + H] ^+^ calcd for 650.2356, found 650.2352.

**2-(benzylamino)-1,3-bis(8-phenylnaphthalen-1-yl)-1,3-dihydrobenzo[d][1,3,2]diazaphosphole 2-oxide (22):** pink solid, 25 mg, 89% yield; [α]_D_^25^ = +82.2 (*c* = 0.013, CHCl_3_).

^1^H NMR (400 MHz, DMSO-D6) δ 8.19 (dd, *J* = 13.9, 7.2 Hz, 3H), 8.12 (d, *J* = 8.2 Hz, 1H), 7.78 (t, *J* = 7.7 Hz, 1H), 7.75 – 7.69 (m, 1H), 7.66 (t, *J* = 7.7 Hz, 1H), 7.61 (t, *J* = 7.6 Hz, 2H), 7.47 (d, *J* = 7.4 Hz, 1H), 7.37 (d, *J* = 15.9 Hz, 1H), 7.26 (dd, *J* = 8.2, 7.5 Hz, 2H), 7.16 (d, *J* = 7.0 Hz, 1H), 7.12 (t, *J* = 7.1 Hz, 1H), 7.06 (t, *J* = 7.4 Hz, 1H), 6.96 – 6.88 (m, 5H), 6.81 – 6.70 (m, 3H), 6.65 (dd, *J* = 16.6, 8.8 Hz, 2H), 6.46 (d, *J* = 7.6 Hz, 2H), 6.37 (d, *J* = 20.9 Hz, 1H), 6.32 (d, *J* = 7.6 Hz, 1H), 5.72 (d, *J* = 7.7 Hz, 1H), 3.68 – 3.52 (m, 2H).

^13^C NMR (101 MHz, DMSO-D6) δ = 143.18, 141.70, 140.63, 140.58, 139.68, 137.60, 136.61, 136.16, 134.85, 134.69, 133.99, 133.81, 132.47, 132.43, 132.30, 132.26, 132.08, 131.69, 130.89, 130.25, 130.17, 129.95, 129.90, 129.86, 129.76, 129.19, 128.82, 128.26, 127.16, 127.07, 126.94, 126.80, 126.57, 126.00, 125.96, 125.74, 119.51, 110.08, 110.00, 109.59, 109.51, 45.83.

^31^P NMR (162 MHz, DMSO-D6) δ = 8.35.

HRMS (ESI- TOF) *m*/*z* [C_45_H_34_N_3_OP + H] ^+^ calcd for 664.2512, found 664.2510.

**2-(naphthalen-1-ylamino)-1,3-bis(8-phenylnaphthalen-1-yl)-1,3-dihydrobenzo[d][1,3,2]diazaphosphole 2-oxide (24):** red brown solid, 24 mg, 86% yield; [α]_D_^25^ = +174.8 (*c* = 0.020, CHCl_3_).

^1^H NMR (400 MHz, CHLOROFORM-D) δ 8.05 (d, *J* = 7.7 Hz, 2H), 7.99 (d, *J* = 8.1 Hz, 1H), 7.84 (d, *J* = 8.0 Hz, 1H), 7.79 (d, *J* = 7.2 Hz, 1H), 7.76 – 7.67 (m, 2H), 7.65 – 7.60 (m, 2H), 7.43 (dt, *J* = 15.9, 7.8 Hz, 3H), 7.36 – 7.30 (m, 2H), 7.29 (s, 2H), 7.24 (d, *J* = 7.5 Hz, 1H), 7.14 (t, *J* = 7.6 Hz, 1H), 7.09 (d, *J* = 6.9 Hz, 1H), 6.97 (t, *J* = 7.4 Hz, 1H), 6.89 (dd, *J* = 17.0, 8.2 Hz, 3H), 6.80 (dd, *J* = 11.6, 7.2 Hz, 3H), 6.73 (t, *J* = 7.5 Hz, 3H), 6.53 (d, *J* = 7.5 Hz, 1H), 6.43 (t, *J* = 7.0 Hz, 1H), 6.33 (d, *J* = 7.4 Hz, 1H), 5.95 (d, *J* = 7.6 Hz, 1H), 3.61 (d, *J* = 7.3 Hz, 1H).

^13^C NMR (101 MHz, CHLOROFORM-D) δ = 142.31, 140.48, 138.59, 136.63, 135.67, 135.04, 134.37, 134.20, 133.44, 133.15, 132.96, 131.76, 131.35, 130.95, 130.80, 130.72, 130.30, 130.11, 129.15, 128.96, 128.84, 128.71, 128.55, 128.40, 128.18, 127.93, 127.85, 127.07, 125.76, 125.48, 125.26, 124.85, 124.73, 124.65, 124.36, 124.23, 124.08, 123.98, 123.57, 123.11, 120.91, 120.74, 118.69, 118.27, 109.40, 108.75.

^31^P NMR (162 MHz, CHLOROFORM-D) δ = 2.32 (s).

HRMS (ESI- TOF) *m*/*z* [C_48_H_34_N_3_OP + H] ^+^ calcd for 700.2512, found 700.2510.

**2-((furan-2-ylmethyl)amino)-1,3-bis(8-phenylnaphthalen-1-yl)-1,3-dihydrobenzo[d][1,3,2]diazaphosphole 2-oxide (19):** pale yellow solid, 24 mg, 84% yield; [α]_D_^25^ = -68.9 (*c* = 0.017, CHCl_3_).

^1^H NMR (400 MHz, DMSO-D6) δ 8.10 (ddd, *J* = 8.2, 7.1, 5.1 Hz, 3H), 8.03 (dd, *J* = 8.2, 1.0 Hz, 1H), 7.71 (t, *J* = 7.7 Hz, 1H), 7.67 – 7.62 (m, 1H), 7.60 – 7.51 (m, 3H), 7.45 (d, *J* = 7.3 Hz, 1H), 7.30 – 7.20 (m, 3H), 7.17 (dd, *J* = 7.0, 1.2 Hz, 1H), 7.14 – 7.08 (m, 2H), 6.90 – 6.82 (m, 3H), 6.62 (t, *J* = 7.4 Hz, 4H), 6.55 (t, *J* = 7.6 Hz, 1H), 6.33 (s, 1H), 6.10 (d, *J* = 7.5 Hz, 1H), 6.07 (dd, *J* = 3.1, 1.9 Hz, 1H), 5.59 (d, *J* = 7.7 Hz, 1H), 5.52 (d, *J* = 3.2 Hz, 1H), 3.67 – 3.46 (m, 2H).

^13^C NMR (101 MHz, DMSO-D6) δ = 153.50, 153.45, 143.19, 142.14, 141.58, 139.52, 137.63, 136.48, 136.09, 134.50, 134.33, 133.91, 133.74, 132.13, 132.09, 132.01, 131.95, 131.81, 130.69, 130.67, 130.23, 130.16, 129.93, 129.76, 129.72, 128.99, 128.57, 127.18, 126.74, 126.67, 126.49, 125.89, 125.68, 119.56, 119.32, 110.56, 109.91, 109.83, 109.56, 109.48, 106.55, 55.45.

^31^P NMR (162 MHz, DMSO-D6) δ = 8.16.

HRMS (ESI- TOF) *m*/*z* [C_43_H_32_N_3_O_2_P + H] ^+^ calcd for 654.2305, found 654.2300.

**2-((2-(benzyloxy)ethyl)amino)-1,3-bis(8-phenylnaphthalen-1-yl)-1,3-dihydrobenzo[d][1,3,2]diazaphosphole 2-oxide (25):** pale yellow solid, 21 mg, 74% yield; [α]_D_^25^ = +75.0 (*c* = 0.033, CHCl_3_).

^1^H NMR (400 MHz, CHLOROFORM-D) δ 7.87 (t, *J* = 7.0 Hz, 3H), 7.80 (d, *J* = 8.0 Hz, 1H), 7.70 (d, *J* = 4.3 Hz, 1H), 7.55 – 7.47 (m, 2H), 7.43 – 7.35 (m, 3H), 7.27 (s, 1H), 7.23 (d, *J* = 6.6 Hz, 2H), 7.20 (s, 1H), 7.18 (s, 1H), 7.14 (d, *J* = 7.0 Hz, 2H), 7.05 (dd, *J* = 12.4, 6.7 Hz, 4H), 6.83 (t, *J* = 7.2 Hz, 2H), 6.57 (dd, *J* = 17.6, 9.9 Hz, 4H), 6.49 (t, *J* = 7.3 Hz, 1H), 6.32 (s, 1H), 6.05 (d, *J* = 7.4 Hz, 1H), 5.65 (d, *J* = 7.4 Hz, 1H), 4.02 (s, 2H), 2.89 – 2.81 (m, 1H), 2.71 (dd, *J* = 11.9, 4.1 Hz, 2H), 2.56 (d, *J* = 5.3 Hz, 1H). ^13^C NMR (101 MHz, CHLOROFORM-D) δ = 143.26, 141.45, 139.83, 138.38, 137.97, 136.35, 136.03, 134.58, 134.41, 134.10, 133.92, 132.51, 132.47, 132.07, 132.04, 131.91, 131.62, 131.48, 131.21, 131.19, 130.43, 130.40, 129.92, 129.87, 129.74, 129.51, 129.49, 129.37, 128.72, 128.38, 128.35, 127.72, 127.67, 126.58, 126.45, 126.24, 125.68, 125.52, 124.91, 124.58, 119.15, 118.96, 109.74, 109.51, 72.96, 71.99, 42.58.

^31^P NMR (162 MHz, CHLOROFORM-D) δ = 8.47.

HRMS (ESI- TOF) *m*/*z* [C_47_H_38_N_3_O_2_P + H] ^+^ calcd for 708.2774, found 708.2764.

1.6 Synthesis and Characterization of **26**.

A dried argon-flushed Schlenk flask was charged with dry THF solution of **17** (0.1 mmol, 0.057 mg), at -78°C, a solution of *n*-BuLi ((0.12 mmol, 0.007 g, 1.6 M in hexane) was added dropwise and stirred for 20 min. Ph_2_PCl (0.2 mmol, 0.044 g) was added dropwise via syringe into the mixture at -78 °C, the resultant mixture was allowed to warm to r.t. and stirred for 6 h. Monitor by TLC plate and dry for column directly (Hexane / Ethyl acetate = 50:1 to 10:1).

**2-((diphenylphosphaneyl)methyl)-1,3-bis(8-phenylnaphthalen-1-yl)-1,3-dihydrobenzo[d][1,3,2]diazaphosphole 2-oxide (26):** pale white solid, 18 mg, 47% yield; [α]_D_^25^ = -20.1 (*c* = 0.007, CHCl_3_).

^1^H NMR (400 MHz, CHLOROFORM-D) δ 7.94 – 7.82 (m, 4H), 7.53 (t, *J* = 7.7 Hz, 1H), 7.47 (t, *J* = 7.5 Hz, 2H), 7.41 – 7.36 (m, 1H), 7.32 (t, *J* = 7.2 Hz, 2H), 7.16 – 7.10 (m, 2H), 7.03 (dd, *J* = 12.2, 6.7 Hz, 4H), 6.96 (t, *J* = 7.1 Hz, 2H), 6.90 (dd, *J* = 14.0, 6.5 Hz, 2H), 6.86 – 6.80 (m, 3H), 6.80 – 6.75 (m, 4H), 6.70 (d, *J* = 7.0 Hz, 2H), 6.64 (d, *J* = 7.2 Hz, 1H), 6.59 – 6.54 (m, 2H), 6.41 (d, *J* = 7.7 Hz, 3H), 5.85 (d, *J* = 7.6 Hz, 1H), 2.13 – 1.96 (m, 2H).

^13^C NMR (126 MHz, cdcl_3_) δ = 142.69, 141.51, 140.01, 137.35, 136.91, 136.58, 135.98, 135.00, 132.72, 132.70, 132.41, 132.21, 131.61, 131.59, 131.51, 131.19, 131.11, 131.07, 131.04, 130.95, 130.94, 130.87, 130.82, 130.80, 130.73, 130.08, 130.04, 129.39, 128.77, 128.63, 128.54, 127.92, 127.59, 127.49, 127.19, 127.10, 126.60, 126.27, 125.98, 125.91, 125.90, 125.75, 124.86, 119.86, 119.62, 111.32, 110.23, 38.69.

^31^P NMR (162 MHz, CHLOROFORM-D) δ = 25.08, 24.76, -26.83, -27.16.

HRMS (ESI- TOF) *m*/*z* [C_51_H_38_N_2_OP_2_ + H] ^+^ calcd for 757.2532, found 773.2480 (+O).

Note: the obtained product may not stable in the air. It should be stored under argon and refrigerator.

2 NMR Spectrum

^1^H NMR

^13^C NMR

^1^H NMR

^13^C NMR

^1^H NMR

^13^C NMR

^31^P NMR

^1^H NMR

^13^C NMR

^31^P NMR

^1^H NMR

^13^C NMR

^31^P NMR

^1^H NMR

^13^C NMR

^31^P NMR

^1^H NMR

^13^C NMR

^31^P NMR

^1^H NMR

^13^C NMR

^31^P NMR

^1^H NMR

^13^C NMR

^31^P NMR

^1^H NMR

^13^C NMR

^31^P NMR

^1^H NMR

^13^C NMR

^31^P NMR

^1^H NMR

^13^C NMR

^31^P NMR

^1^H NMR

^13^C NMR

^31^P NMR

^1^H NMR

^13^C NMR

^31^P NMR

^1^H NMR

^13^C NMR

^31^P NMR

^1^H NMR

^13^C NMR

^31^P NMR

3 HPLC Chromatograms

**Figure S1**. HPLC spectrum of racemic **11**.

**Figure S2**. HPLC spectrum of isomer **11a**.

**Figure S3**. HPLC spectrum of isomer **11b**.

**Figure S4**. HPLC spectrum of racemic **15**.

**Figure S5**. HPLC spectrum of isomer **15a**.

**Figure S6**. HPLC spectrum of isomer **15b**.

**Figure S7**. HPLC spectrum of racemic **16**.

**Figure S8**. HPLC spectrum of isomer **16a**.

**Figure S9**. HPLC spectrum of isomer **16b**.

**Figure S10**. HPLC spectrum of racemic **17**.

**Figure S11**. HPLC spectrum of isomer **17**.

4 X-ray Data of 9 & 15a
  4.1 General Data Collection

Data were collected on a Bruker PLATFORM three circle diffractometer equipped with an APEX II CCD detector and operated at 1350 W (40kV, 30 mA) to generate (graphite monochromated) Mo Kα radiation (λ = 0.71073 Å). Crystals were transferred from the vial and placed on a glass slide in polyisobutylene. A Zeiss Stemi 305 microscope was used to identify a suitable specimen for X-ray diffraction from a representative sample of the material. The crystal and a small amount of the oil were collected on a MῑTiGen cryoloop and transferred to the instrument where it was placed under a cold nitrogen stream (Oxford) maintained at 100K throughout the duration of the experiment. The samples were optically centered with the aid of a video camera to insure that no translations were observed as the crystal was rotated through all positions.

A unit cell collection was then carried out. After it was determined that the unit cell was not present in the CCDC database a sphere of data was collected. Omega scans were carried out with a 120 sec/frame exposure time and a rotation of 2.0° per frame. After data collection, the crystal was measured for size, morphology, and color. These values are reported in Table S1 & S6.

Refinement Details

After data collection, the unit cell was re-determined using a subset of the full data collection. Intensity data were corrected for Lorentz, polarization, and background effects using the Bruker program APEX 3. A semi-empirical correction for adsorption was applied using the program *SADABS* [62]. The *SHELXL-2014* [63], series of programs was used for the solution and refinement of the crystal structure. Hydrogen atoms bound to carbon and nitrogen atoms were located in the difference Fourier map and were geometrically constrained using the appropriate AFIX commands. Due to poor crystal diffraction quality of the crystals the rigid-bond restraint RIGU was applied globally during the final refinements.

**Figure S12.** X-ray structure of **9**.

The thermal ellipsoids are represented at 50% probability. Carbon, hydrogen, and nitrogen atoms are represented by gray, white, and light blue ellipsoids, respectively.

**Figure S13.** Crystal used for single crystal X-ray diffraction analysis.

**Table S1.** Crystal data and structure refinement for **9**.

Identification code	li18_11

Crystal Color	green

Crystal Habit	blocky

Empirical formula	C38 H28 N2

Formula weight	512.62

Temperature	100(2) K

Wavelength	0.71073 A

Crystal system	Hexagonal

Space group	P6_1_

Unit cell dimensions	a = 9.8935(4) Å	alpha = 90 °.

b = 9.8935(4) Å	beta = 90 °.

c = 47.264(2) Å	gamma = 120 °.

Volume	4006.5(4) Å^3^

Z	6

Calculated density	1.275 Mg/m^3^

Absorption coefficient	0.074 mm^-1^

F(000)	1620

Crystal size	0.320 x 0.225 x 0.080 mm

Theta range for data collection	2.377 to 25.426 °.

Limiting indices	-11<=h<=8, -10<=k<=11, -57<=l<=56

Reflections collected / unique	27427 / 4865 [R(int) = 0.0788]

Completeness to theta = 25.242°	100.0 %

Refinement method	Full-matrix least-squares on F^2^

Data / restraints / parameters	4865 / 331 / 362

Goodness-of-fit on F^2^	1.027

Final R indices [I>2sigma(I)]	R1 = 0.0631, wR2 = 0.1261

R indices (all data)	R1 = 0.1224, wR2 = 0.1493

Absolute structure parameter	-2.1(10)

Extinction coefficient	0.0146(17)

Largest diff. peak and hole	0.241 and -0.200 e.A^-3^

**Table S2.** Atomic coordinates ( x 10^4^) and equivalent isotropic displacement parameters (Å^2^ x 10^3^) for **9**. U(eq) is defined as one third of the trace of the orthogonalized Uij tensor.

```
_______________________________________________________________________
                         x             y             z           U(eq)

          N(1)         5422(6)       3663(6)       5240(1)       42(1)
          N(2)         6423(6)       5788(5)       4813(1)       41(1)
          C(1)         4378(6)       4090(6)       5127(1)       29(1)
          C(2)         4941(7)       5268(7)       4924(1)       32(1)
          C(3)         3967(7)       5820(7)       4834(1)       42(2)
          C(4)         2469(8)       5195(7)       4935(2)       46(2)
          C(5)         1896(7)       4000(7)       5129(1)       44(2)
          C(6)         2843(7)       3439(7)       5224(1)       38(2)
          C(7)         5220(7)       2174(6)       5251(1)       32(1)
          C(8)         6268(7)       1877(7)       5412(1)       32(1)
          C(9)         7596(7)       3029(7)       5569(1)       37(2)
          C(10)        8430(8)       2541(9)       5731(1)       55(2)
          C(11)        8078(10)      1015(9)       5746(2)       62(2)
          C(12)        6855(8)        -99(9)       5595(1)       51(2)
          C(13)        5945(7)        306(7)       5426(1)       37(2)
          C(14)        4664(7)       -893(7)       5275(1)       44(2)
          C(15)        3726(7)       -550(8)       5118(1)       45(2)
          C(16)        4002(7)        968(7)       5105(1)       41(2)
          C(17)        8159(7)       4748(7)       5565(1)       32(1)
          C(18)        9171(7)       5723(8)       5360(1)       42(2)
          C(19)        9738(7)       7313(8)       5365(2)       48(2)
          C(20)        9334(7)       7958(8)       5582(2)       45(2)
          C(21)        8339(8)       7026(8)       5788(1)       47(2)
          C(22)        7772(7)       5440(8)       5781(1)       39(2)
          C(23)        7630(7)       7315(7)       4779(1)       33(1)
          C(24)        8988(7)       7638(7)       4613(1)       30(1)
          C(25)        9193(6)       6481(7)       4465(1)       32(1)
          C(26)       10560(7)       6923(7)       4324(1)       45(2)
          C(27)       11744(7)       8480(8)       4310(1)       48(2)
          C(28)       11593(7)       9585(7)       4447(1)       39(2)
          C(29)       10237(7)       9205(6)       4602(1)       30(1)
          C(30)       10105(7)      10394(7)       4747(1)       38(2)
          C(31)        8804(7)      10032(7)       4901(1)       40(2)
          C(32)        7577(8)       8503(7)       4915(1)       38(2)
          C(33)        7993(7)       4791(7)       4441(1)       33(1)
          C(34)        6822(7)       4329(8)       4238(2)       50(2)
          C(35)        5764(8)       2765(8)       4201(2)       56(2)
          C(36)        5853(9)       1660(8)       4360(2)       64(2)
          C(37)        7021(8)       2106(8)       4566(2)       57(2)
          C(38)        8081(7)       3663(8)       4598(2)       48(2)
_______________________________________________________________________
```

**Table S3.** Bond lengths [Å] and angles [°] for **9**.

```
_____________________________________________________________

          N(1)         5422(6)       3663(6)       5240(1)       42(1)
          N(2)         6423(6)       5788(5)       4813(1)       41(1)
          C(1)         4378(6)       4090(6)       5127(1)       29(1)
          C(2)         4941(7)       5268(7)       4924(1)       32(1)
          C(3)         3967(7)       5820(7)       4834(1)       42(2)
          C(4)         2469(8)       5195(7)       4935(2)       46(2)
          C(5)         1896(7)       4000(7)       5129(1)       44(2)
          C(6)         2843(7)       3439(7)       5224(1)       38(2)
          C(7)         5220(7)       2174(6)       5251(1)       32(1)
          C(8)         6268(7)       1877(7)       5412(1)       32(1)
          C(9)         7596(7)       3029(7)       5569(1)       37(2)
          C(10)        8430(8)       2541(9)       5731(1)       55(2)
          C(11)        8078(10)      1015(9)       5746(2)       62(2)
          C(12)        6855(8)        -99(9)       5595(1)       51(2)
          C(13)        5945(7)        306(7)       5426(1)       37(2)
          C(14)        4664(7)       -893(7)       5275(1)       44(2)
          C(15)        3726(7)       -550(8)       5118(1)       45(2)
          C(16)        4002(7)        968(7)       5105(1)       41(2)
          C(17)        8159(7)       4748(7)       5565(1)       32(1)
          C(18)        9171(7)       5723(8)       5360(1)       42(2)
          C(19)        9738(7)       7313(8)       5365(2)       48(2)
          C(20)        9334(7)       7958(8)       5582(2)       45(2)
          C(21)        8339(8)       7026(8)       5788(1)       47(2)
          C(22)        7772(7)       5440(8)       5781(1)       39(2)
          C(23)        7630(7)       7315(7)       4779(1)       33(1)
          C(24)        8988(7)       7638(7)       4613(1)       30(1)
          C(25)        9193(6)       6481(7)       4465(1)       32(1)
          C(26)       10560(7)       6923(7)       4324(1)       45(2)
          C(27)       11744(7)       8480(8)       4310(1)       48(2)
          C(28)       11593(7)       9585(7)       4447(1)       39(2)
          C(29)       10237(7)       9205(6)       4602(1)       30(1)
          C(30)       10105(7)      10394(7)       4747(1)       38(2)
          C(31)        8804(7)      10032(7)       4901(1)       40(2)
          C(32)        7577(8)       8503(7)       4915(1)       38(2)
          C(33)        7993(7)       4791(7)       4441(1)       33(1)
          C(34)        6822(7)       4329(8)       4238(2)       50(2)
          C(35)        5764(8)       2765(8)       4201(2)       56(2)
          C(36)        5853(9)       1660(8)       4360(2)       64(2)
          C(37)        7021(8)       2106(8)       4566(2)       57(2)
          C(38)        8081(7)       3663(8)       4598(2)       48(2)
_____________________________________________________________
```

Symmetry transformations used to generate equivalent atoms:

**Table S4.** Anisotropic displacement parameters (Å^2^ x 10^3^) for **9**. The anisotropic displacement factor exponent takes the form: -2 π^2^ [ h^2^ a*^2^U^11^ + ... + 2 h k a* b* U^12^ ]

```
_________________________________________________________________________
              U11        U22        U33        U23        U13        U12
_________________________________________________________________________

    N(1)     32(3)      33(3)      59(4)       6(3)     -13(3)      14(3)
    N(2)     42(3)      29(3)      58(4)       8(3)      21(3)      24(3)
    C(1)     28(3)      28(3)      32(3)      -5(3)      -7(3)      14(3)
    C(2)     31(3)      33(4)      37(3)       3(3)       6(3)      19(3)
    C(3)     45(4)      40(4)      49(4)      11(3)       2(3)      27(3)
    C(4)     42(4)      41(4)      68(5)      -8(3)      -7(3)      30(4)
    C(5)     28(4)      38(4)      61(4)     -12(3)       0(3)      12(3)
    C(6)     28(3)      36(4)      41(4)      -1(3)       5(3)      10(3)
    C(7)     25(3)      30(3)      38(3)       5(3)       3(3)      13(3)
    C(8)     38(3)      38(3)      25(3)       2(3)       5(3)      24(3)
    C(9)     46(4)      46(4)      30(3)       2(3)       1(3)      32(3)
    C(10)    67(5)      61(5)      53(4)      -8(4)     -18(4)      44(4)
    C(11)    92(6)      67(5)      60(5)      -9(4)     -26(4)      64(5)
    C(12)    67(5)      51(4)      46(4)       3(3)       0(4)      39(4)
    C(13)    40(4)      36(3)      41(4)       3(3)      11(3)      24(3)
    C(14)    35(4)      33(4)      65(5)       0(3)      13(3)      19(3)
    C(15)    29(4)      39(4)      65(5)     -12(3)      -1(3)      14(3)
    C(16)    26(3)      35(4)      59(4)      -3(3)       0(3)      13(3)
    C(17)    34(4)      44(4)      23(3)      -2(3)      -5(3)      23(3)
    C(18)    43(4)      53(4)      36(4)       7(3)       7(3)      29(3)
    C(19)    27(4)      56(4)      59(4)       7(4)      -2(3)      19(3)
    C(20)    43(4)      35(4)      51(4)      -4(3)     -16(3)      16(3)
    C(21)    59(5)      50(4)      40(4)      -5(3)      -2(3)      33(4)
    C(22)    43(4)      49(4)      32(3)       0(3)      -1(3)      27(3)
    C(23)    34(3)      34(3)      35(3)       1(3)       0(3)      20(3)
    C(24)    33(3)      35(3)      22(3)       0(3)      -2(3)      18(3)
    C(25)    27(3)      34(3)      35(3)       1(3)      -1(3)      15(3)
    C(26)    37(4)      39(4)      50(4)     -10(3)       8(3)      12(3)
    C(27)    30(4)      45(4)      53(4)      -9(3)      11(3)       7(3)
    C(28)    32(4)      37(4)      32(3)       0(3)       2(3)       4(3)
    C(29)    30(3)      31(3)      29(3)       0(3)      -5(3)      14(3)
    C(30)    37(4)      36(4)      40(4)      -3(3)      -9(3)      17(3)
    C(31)    39(4)      36(4)      47(4)      -8(3)      -8(3)      22(3)
    C(32)    42(4)      43(4)      33(3)      -2(3)       1(3)      24(3)
    C(33)    26(3)      36(3)      36(3)      -3(3)       2(3)      15(3)
    C(34)    42(4)      47(4)      44(4)       3(3)      -9(3)      11(3)
    C(35)    39(4)      47(4)      58(4)      -5(4)     -11(4)       3(4)
    C(36)    40(4)      33(4)     108(7)     -13(4)      -4(4)      10(4)
    C(37)    36(4)      35(4)     101(6)       8(4)       0(4)      17(3)
    C(38)    33(4)      39(4)      72(5)       5(3)      -4(3)      18(3)
_________________________________________________________________________
```

**Table S5.** Hydrogen coordinates (x 10^4^) and isotropic displacement parameters (Å^2^ x 10^3^) for **9**.

________________________________________________________________

```
                          x             y             z           U(eq)
________________________________________________________________
          H(1)         6297          4420          5311          51
          H(2)         6622          5056          4757          49
          H(3)         4345          6646          4700          50
          H(4)         1825          5590          4870          55
          H(5)          858          3566          5198          53
          H(6)         2449          2605          5356          45
          H(10)        9290          3299          5838          66
          H(11)        8685           735          5862          75
          H(12)        6617         -1155          5604          61
          H(14)        4464         -1937          5284          52
          H(15)        2878         -1354          5015          54
          H(16)        3337          1186          4993          50
          H(18)        9483          5290          5211          50
          H(19)       10409          7958          5219          58
          H(20)        9746          9054          5589          54
          H(21)        8036          7469          5936          57
          H(22)        7100          4805          5928          47
          H(26)       10706          6148          4234          54
          H(27)       12659          8758          4202          58
          H(28)       12415         10639          4439          47
          H(30)       10928         11446          4736          46
          H(31)        8735         10832          4999          47
          H(32)        6677          8280          5022          45
          H(34)        6752          5088          4126          60
          H(35)        4962          2454          4064          67
          H(36)        5122           586          4330          77
          H(37)        7077          1346          4680          69
          H(38)        8899          3973          4732          58
        ________________________________________________________________
```

**Figure S14.** X-ray structure of **15a**.

The thermal ellipsoids are represented at 50% probability. Carbon, hydrogen, nitrogen, oxygen, and phosphorus atoms are represented by gray, white, light blue, red, and light orange ellipsoids, respectively.

**Figure S15.** Crystal used for single crystal X-ray diffraction analysis.

**Table S6.** Crystal data and structure refinement for **15a**.

Identification code	li18_12

Crystal Color	colorless

Crystal Habit	plate

Empirical formula	C38 H28 N3 O P

Formula weight	573.60

Temperature	100(2) K

Wavelength	0.71073 Å

Crystal system	Orthorhombic

Space group	*P*2_1_2_1_2_1_

Unit cell dimensions	a = 9.361(3) Å	alpha = 90 °.

b = 12.077(4) Å	beta = 90 °.

c = 25.278(10) Å	gamma = 90 °.

Volume	2857.5(18) Å^3^

Z	4

Calculated density	1.333 Mg/m^3^

Absorption coefficient	0.134 mm^-1^

F(000)	1200

Crystal size	0.270 x 0.081 x 0.015 mm

Theta range for data collection	1.869 to 25.431 °.

Limiting indices	-11<=h<=9, -14<=k<=13, -30<=l<=30

Reflections collected / unique	15101 / 5123 [R(int) = 0.0942]

Completeness to theta = 25.242°	99.5 %

Refinement method	Full-matrix least-squares on F^2^

Data / restraints / parameters	5123 / 0 / 388

Goodness-of-fit on F^2^	0.927

Final R indices [I>2sigma(I)]	R1 = 0.0580, wR2 = 0.0873

R indices (all data)	R1 = 0.1252, wR2 = 0.1080

Absolute structure parameter	-0.08(15)

Largest diff. peak and hole	0.262 and -0.307 e.Å^-3^

**Table S7.** Atomic coordinates ( x 10^4^) and equivalent isotropic displacement parameters (Å^2^ x 10^3^) for **15a**. U(eq) is defined as one third of the trace of the orthogonalized Uij tensor.

```
   	   _______________________________________________________________________
                         x             y             z           U(eq)

          P(1)         2224(2)       2979(1)       5687(1)       20(1)
          O(1)         3010(4)       2184(3)       5358(1)       21(1)
          N(1)         2916(5)       4261(3)       5734(2)       16(1)
          N(2)         2268(5)       2819(3)       6349(2)       16(1)
          N(3)          561(4)       3041(4)       5503(2)       22(1)
          C(1)         3341(6)       4532(5)       6251(2)       19(2)
          C(2)         4129(6)       5422(5)       6416(2)       24(2)
          C(3)         4351(6)       5572(5)       6958(2)       27(2)
          C(4)         3768(6)       4838(5)       7317(2)       27(2)
          C(5)         3029(6)       3909(5)       7146(2)       23(2)
          C(6)         2856(6)       3739(4)       6611(2)       17(1)
          C(7)         3544(6)       4810(4)       5280(2)       18(1)
          C(8)         2821(7)       5632(4)       4978(2)       20(1)
          C(9)         1409(7)       6081(5)       5076(2)       21(2)
          C(10)         829(6)       6806(5)       4718(2)       27(2)
          C(11)        1558(7)       7147(5)       4261(2)       32(2)
          C(12)        2888(7)       6765(4)       4169(2)       28(2)
          C(13)        3565(7)       6014(5)       4521(2)       23(2)
          C(14)        4964(6)       5642(5)       4403(2)       26(2)
          C(15)        5632(6)       4901(5)       4713(2)       25(2)
          C(16)        4905(6)       4474(5)       5156(2)       22(2)
          C(17)         525(7)       5877(4)       5559(2)       23(2)
          C(18)         972(6)       6231(5)       6051(2)       25(2)
          C(19)         127(7)       6082(5)       6492(2)       34(2)
          C(20)       -1223(7)       5605(5)       6443(3)       37(2)
          C(21)       -1715(7)       5316(5)       5948(3)       37(2)
          C(22)        -862(7)       5446(5)       5511(2)       28(2)
          C(23)        1546(6)       1955(5)       6634(2)       19(1)
          C(24)        2126(7)        864(4)       6689(2)       17(1)
          C(25)        3524(6)        498(5)       6532(2)       20(2)
          C(26)        3851(7)       -610(5)       6554(2)       24(2)
          C(27)        2917(7)      -1401(5)       6759(2)       33(2)
          C(28)        1627(7)      -1072(5)       6953(2)       31(2)
          C(29)        1203(6)         43(5)       6923(2)       22(2)
          C(30)        -159(7)        349(5)       7113(2)       28(2)
          C(31)        -639(7)       1400(6)       7075(2)       30(2)
          C(32)         218(6)       2207(5)       6827(2)       26(2)
          C(33)        4741(6)       1262(5)       6400(2)       19(2)
          C(34)        5157(6)       2069(5)       6755(2)       22(2)
          C(35)        6309(6)       2746(5)       6646(2)       29(2)
          C(36)        7087(7)       2594(5)       6189(2)       28(2)
          C(37)        6718(6)       1755(5)       5843(2)       30(2)
          C(38)        5534(7)       1102(5)       5942(2)       29(2)
_______________________________________________________________________
```

**Table S8.** Bond lengths [Å] and angles [°] for **15a**.

```
_____________________________________________________________

            P(1)-O(1)                     1.468(3)
            P(1)-N(3)                     1.626(4)
            P(1)-N(1)                     1.682(4)
            P(1)-N(2)                     1.685(4)
            N(1)-C(1)                     1.406(6)
            N(1)-C(7)                     1.449(6)
            N(2)-C(6)                     1.405(6)
            N(2)-C(23)                    1.438(6)
            N(3)-H(3A)                    0.8800
            N(3)-H(3B)                    0.8800
            C(1)-C(2)                     1.369(7)
            C(1)-C(6)                     1.396(7)
            C(2)-C(3)                     1.399(7)
            C(2)-H(2)                     0.9500
            C(3)-C(4)                     1.381(7)
            C(3)-H(3)                     0.9500
            C(4)-C(5)                     1.387(7)
            C(4)-H(4)                     0.9500
            C(5)-C(6)                     1.378(7)
            C(5)-H(5)                     0.9500
            C(7)-C(16)                    1.373(7)
            C(7)-C(8)                     1.423(7)
            C(8)-C(13)                    1.426(7)
            C(8)-C(9)                     1.450(8)
            C(9)-C(10)                    1.371(7)
            C(9)-C(17)                    1.495(8)
            C(10)-C(11)                   1.404(7)
            C(10)-H(10)                   0.9500
            C(11)-C(12)                   1.347(7)
            C(11)-H(11)                   0.9500
            C(12)-C(13)                   1.420(7)
            C(12)-H(12)                   0.9500
            C(13)-C(14)                   1.416(8)
            C(14)-C(15)                   1.344(7)
            C(14)-H(14)                   0.9500
            C(15)-C(16)                   1.408(7)
            C(15)-H(15)                   0.9500
            C(16)-H(16)                   0.9500
            C(17)-C(18)                   1.380(7)
            C(17)-C(22)                   1.404(8)
            C(18)-C(19)                   1.377(8)
            C(18)-H(18)                   0.9500
            C(19)-C(20)                   1.395(8)
            C(19)-H(19)                   0.9500
            C(20)-C(21)                   1.378(8)
            C(20)-H(20)                   0.9500
            C(21)-C(22)                   1.372(8)
            C(21)-H(21)                   0.9500
            C(22)-H(22)                   0.9500
            C(23)-C(32)                   1.369(7)
            C(23)-C(24)                   1.433(7)
            C(24)-C(25)                   1.437(7)
            C(24)-C(29)                   1.442(7)
            C(25)-C(26)                   1.374(7)
            C(25)-C(33)                   1.503(7)
            C(26)-C(27)                   1.394(7)
            C(26)-H(26)                   0.9500
            C(27)-C(28)                   1.363(8)
            C(27)-H(27)                   0.9500
            C(28)-C(29)                   1.405(7)
            C(28)-H(28)                   0.9500
            C(29)-C(30)                   1.412(7)
            C(30)-C(31)                   1.349(8)
            C(30)-H(30)                   0.9500
            C(31)-C(32)                   1.409(7)
            C(31)-H(31)                   0.9500
            C(32)-H(32)                   0.9500
            C(33)-C(34)                   1.381(7)
            C(33)-C(38)                   1.389(7)
            C(34)-C(35)                   1.380(7)
            C(34)-H(34)                   0.9500
            C(35)-C(36)                   1.377(7)
            C(35)-H(35)                   0.9500
            C(36)-C(37)                   1.383(7)
            C(36)-H(36)                   0.9500
            C(37)-C(38)                   1.383(7)
            C(37)-H(37)                   0.9500
            C(38)-H(38)                   0.9500

            O(1)-P(1)-N(3)              110.4(2)
            O(1)-P(1)-N(1)              116.7(2)
            N(3)-P(1)-N(1)              110.3(2)
            O(1)-P(1)-N(2)              118.4(2)
            N(3)-P(1)-N(2)              108.2(2)
            N(1)-P(1)-N(2)               91.5(2)
            C(1)-N(1)-C(7)              120.9(4)
            C(1)-N(1)-P(1)              112.9(3)
            C(7)-N(1)-P(1)              121.5(3)
            C(6)-N(2)-C(23)             121.4(4)
            C(6)-N(2)-P(1)              112.8(3)
            C(23)-N(2)-P(1)             124.7(3)
            P(1)-N(3)-H(3A)             120.0
            P(1)-N(3)-H(3B)             120.0
            H(3A)-N(3)-H(3B)            120.0
            C(2)-C(1)-C(6)              121.1(5)
            C(2)-C(1)-N(1)              128.2(5)
            C(6)-C(1)-N(1)              110.7(5)
            C(1)-C(2)-C(3)              118.6(6)
            C(1)-C(2)-H(2)              120.7
            C(3)-C(2)-H(2)              120.7
            C(4)-C(3)-C(2)              120.2(6)
            C(4)-C(3)-H(3)              119.9
            C(2)-C(3)-H(3)              119.9
            C(3)-C(4)-C(5)              120.7(5)
            C(3)-C(4)-H(4)              119.6
            C(5)-C(4)-H(4)              119.6
            C(6)-C(5)-C(4)              119.0(5)
            C(6)-C(5)-H(5)              120.5
            C(4)-C(5)-H(5)              120.5
            C(5)-C(6)-C(1)              119.9(5)
            C(5)-C(6)-N(2)              128.8(5)
            C(1)-C(6)-N(2)              111.3(4)
            C(16)-C(7)-C(8)             121.6(5)
            C(16)-C(7)-N(1)             115.0(5)
            C(8)-C(7)-N(1)              123.4(5)
            C(7)-C(8)-C(13)             115.4(5)
            C(7)-C(8)-C(9)              127.1(5)
            C(13)-C(8)-C(9)             117.5(5)
            C(10)-C(9)-C(8)             119.1(5)
            C(10)-C(9)-C(17)            115.2(5)
            C(8)-C(9)-C(17)             125.6(5)
            C(9)-C(10)-C(11)            122.6(6)
            C(9)-C(10)-H(10)            118.7
            C(11)-C(10)-H(10)           118.7
            C(12)-C(11)-C(10)           119.4(6)
            C(12)-C(11)-H(11)           120.3
            C(10)-C(11)-H(11)           120.3
            C(11)-C(12)-C(13)           121.5(6)
            C(11)-C(12)-H(12)           119.2
            C(13)-C(12)-H(12)           119.2
            C(14)-C(13)-C(12)           118.9(6)
            C(14)-C(13)-C(8)            121.3(6)
            C(12)-C(13)-C(8)            119.8(6)
            C(15)-C(14)-C(13)           121.3(6)
            C(15)-C(14)-H(14)           119.3
            C(13)-C(14)-H(14)           119.3
            C(14)-C(15)-C(16)           118.8(6)
            C(14)-C(15)-H(15)           120.6
            C(16)-C(15)-H(15)           120.6
            C(7)-C(16)-C(15)            121.5(5)
            C(7)-C(16)-H(16)            119.2
            C(15)-C(16)-H(16)           119.2
            C(18)-C(17)-C(22)           118.3(6)
            C(18)-C(17)-C(9)            121.1(5)
            C(22)-C(17)-C(9)            120.1(5)
            C(19)-C(18)-C(17)           121.0(6)
            C(19)-C(18)-H(18)           119.5
            C(17)-C(18)-H(18)           119.5
            C(18)-C(19)-C(20)           120.1(6)
            C(18)-C(19)-H(19)           119.9
            C(20)-C(19)-H(19)           119.9
            C(21)-C(20)-C(19)           119.2(6)
            C(21)-C(20)-H(20)           120.4
            C(19)-C(20)-H(20)           120.4
            C(22)-C(21)-C(20)           120.5(6)
            C(22)-C(21)-H(21)           119.8
            C(20)-C(21)-H(21)           119.8
            C(21)-C(22)-C(17)           120.7(6)
            C(21)-C(22)-H(22)           119.7
            C(17)-C(22)-H(22)           119.7
            C(32)-C(23)-C(24)           120.9(5)
            C(32)-C(23)-N(2)            116.4(5)
            C(24)-C(23)-N(2)            122.6(5)
            C(23)-C(24)-C(25)           127.0(5)
            C(23)-C(24)-C(29)           116.4(5)
            C(25)-C(24)-C(29)           116.6(5)
            C(26)-C(25)-C(24)           119.4(5)
            C(26)-C(25)-C(33)           115.9(5)
            C(24)-C(25)-C(33)           124.3(5)
            C(25)-C(26)-C(27)           122.8(6)
            C(25)-C(26)-H(26)           118.6
            C(27)-C(26)-H(26)           118.6
            C(28)-C(27)-C(26)           119.3(6)
            C(28)-C(27)-H(27)           120.4
            C(26)-C(27)-H(27)           120.4
            C(27)-C(28)-C(29)           120.7(6)
            C(27)-C(28)-H(28)           119.7
            C(29)-C(28)-H(28)           119.7
            C(28)-C(29)-C(30)           119.2(6)
            C(28)-C(29)-C(24)           120.8(6)
            C(30)-C(29)-C(24)           120.0(6)
            C(31)-C(30)-C(29)           121.5(6)
            C(31)-C(30)-H(30)           119.2
            C(29)-C(30)-H(30)           119.2
            C(30)-C(31)-C(32)           119.5(6)
            C(30)-C(31)-H(31)           120.2
            C(32)-C(31)-H(31)           120.2
            C(23)-C(32)-C(31)           121.4(6)
            C(23)-C(32)-H(32)           119.3
            C(31)-C(32)-H(32)           119.3
            C(34)-C(33)-C(38)           119.3(6)
            C(34)-C(33)-C(25)           120.2(5)
            C(38)-C(33)-C(25)           120.3(5)
            C(35)-C(34)-C(33)           120.6(6)
            C(35)-C(34)-H(34)           119.7
            C(33)-C(34)-H(34)           119.7
            C(36)-C(35)-C(34)           120.1(6)
            C(36)-C(35)-H(35)           119.9
            C(34)-C(35)-H(35)           119.9
            C(35)-C(36)-C(37)           119.7(6)
            C(35)-C(36)-H(36)           120.1
            C(37)-C(36)-H(36)           120.1
            C(36)-C(37)-C(38)           120.2(6)
            C(36)-C(37)-H(37)           119.9
            C(38)-C(37)-H(37)           119.9
            C(37)-C(38)-C(33)           120.0(6)
            C(37)-C(38)-H(38)           120.0
            C(33)-C(38)-H(38)           120.0
_____________________________________________________________
```

Symmetry transformations used to generate equivalent atoms:

**Table S9.** Anisotropic displacement parameters (Å^2^ x 10^3^) for **15a**. The anisotropic displacement factor exponent takes the form: -2 π^2^ [ h^2^ a*^2^U^11^ + ... + 2 h k a* b* U^12^ ]

```
_________________________________________________________________________
              U11        U22        U33        U23        U13        U12
_________________________________________________________________________

    P(1)     20(1)      19(1)      21(1)       2(1)       0(1)      -1(1)
    O(1)     24(2)      18(2)      20(2)      -3(2)       3(2)       4(2)
    N(1)     15(3)      12(3)      20(2)       1(2)       0(2)      -3(2)
    N(2)     15(3)      15(3)      17(2)       2(2)       3(2)      -5(2)
    N(3)     13(3)      24(3)      27(3)      -5(2)      -3(2)       4(2)
    C(1)     20(4)      19(3)      19(3)      -2(3)      -2(3)       3(3)
    C(2)     24(4)      19(4)      29(4)       1(3)      -3(3)      -2(3)
    C(3)     18(4)      27(4)      35(4)      -5(3)      -7(3)       2(3)
    C(4)     24(4)      32(4)      24(4)      -5(3)      -4(3)       4(3)
    C(5)     24(4)      22(4)      23(3)      -3(3)       3(3)       4(3)
    C(6)      7(3)      20(3)      23(3)      -2(3)      -1(3)       4(3)
    C(7)     16(4)      13(3)      24(3)      -1(3)       0(3)      -2(3)
    C(8)     22(4)      13(3)      24(3)       1(3)      -4(3)      -2(3)
    C(9)     21(4)      16(4)      27(3)      -2(3)      -3(3)      -4(3)
    C(10)    23(4)      25(4)      34(4)      -1(3)      -4(3)       5(3)
    C(11)    39(4)      24(4)      32(4)       5(3)      -4(4)       4(4)
    C(12)    35(4)      22(4)      27(3)       7(3)      -1(3)       3(3)
    C(13)    26(4)      17(3)      26(3)       1(3)      -3(3)      -8(3)
    C(14)    30(4)      20(4)      27(4)       0(3)       4(3)      -6(3)
    C(15)    21(4)      22(4)      33(4)      -1(3)       5(3)      -6(3)
    C(16)    25(4)      17(3)      25(3)       0(3)      -7(3)       0(3)
    C(17)    25(4)      14(3)      31(4)       0(3)      -4(3)       6(3)
    C(18)    23(4)      25(4)      26(3)       1(3)       1(3)       4(3)
    C(19)    40(5)      31(4)      30(4)      -2(3)      -1(4)       9(4)
    C(20)    38(5)      24(4)      49(5)      14(4)      16(4)       6(4)
    C(21)    22(4)      29(4)      59(5)       3(4)       5(4)      -1(3)
    C(22)    26(4)      19(4)      38(4)      -2(3)       1(4)       2(3)
    C(23)    11(4)      21(4)      23(3)      -3(3)      -3(3)      -3(3)
    C(24)    21(4)      18(3)      13(3)      -2(3)      -2(3)       1(3)
    C(25)    21(4)      20(4)      18(3)      -1(3)      -2(3)      -4(3)
    C(26)    27(4)      20(4)      26(3)      -6(3)      -1(3)       1(3)
    C(27)    36(5)      23(4)      39(4)       0(3)      -5(4)       2(4)
    C(28)    37(5)      22(4)      33(4)       7(3)      -8(4)     -14(4)
    C(29)    25(4)      22(4)      20(3)       7(3)      -2(3)      -7(3)
    C(30)    26(4)      39(5)      20(3)       7(3)       3(3)      -8(4)
    C(31)    24(4)      43(5)      24(4)       7(3)      -1(3)      -1(4)
    C(32)    26(4)      28(4)      25(3)       3(3)      -3(3)       2(3)
    C(33)    17(4)      21(4)      19(3)       8(3)      -1(3)       4(3)
    C(34)    22(4)      18(4)      25(3)       1(3)      -1(3)       7(3)
    C(35)    21(4)      29(4)      35(4)       1(3)      -6(3)       3(3)
    C(36)    19(4)      29(4)      37(4)       8(3)       0(3)       2(3)
    C(37)    18(4)      46(5)      26(4)       8(3)       6(3)       7(4)
    C(38)    27(4)      33(4)      27(4)      -3(3)      -3(3)       3(3)

_________________________________________________________________________
```

**Table S10.** Hydrogen coordinates (x 10^4^) and isotropic displacement parameters (Å^2^ x 10^3^) for **15a**.

________________________________________________________________

```
                          x             y             z           U(eq)
________________________________________________________________
          H(3A)         257          2628          5240          26
          H(3B)         -34          3487          5668          26
          H(2)         4518          5929          6166          29
          H(3)         4905          6180          7080          32
          H(4)         3875          4970          7686          32
          H(5)         2646          3398          7394          27
          H(10)        -103          7088          4782          33
          H(11)        1118          7641          4018          38
          H(12)        3385          7005          3862          33
          H(14)        5439          5922          4100          31
          H(15)        6579          4669          4634          30
          H(16)        5366          3942          5373          27
          H(18)        1875          6582          6087          30
          H(19)         464          6306          6830          40
          H(20)       -1797          5480          6747          45
          H(21)       -2652          5024          5909          44
          H(22)       -1214          5242          5172          34
          H(26)        4753          -848          6425          29
          H(27)        3177         -2162          6763          39
          H(28)        1008         -1601          7111          37
          H(30)        -752          -197          7271          34
          H(31)       -1547          1594          7214          37
          H(32)        -134          2941          6792          32
          H(34)        4647          2159          7077          26
          H(35)        6564          3317          6886          34
          H(36)        7873          3065          6112          34
          H(37)        7280          1627          5536          36
          H(38)        5263           544          5697          35
        ________________________________________________________________
```

**Table S11.** Hydrogen bonds for **15a** where hydrogen bonds with H..A < r(A) + 2.000 Å and <DHA > 110° are listed.

```
__________________________________________________________________________
D-H...A                      d(D-H)      d(H...A)    d(D...A)    <(DHA)

N(3)-H(3A)...O(1)#1          0.88        2.60        3.243(5)    130.8
__________________________________________________________________________
```

Symmetry transformations used to generate equivalent atoms:

#1 x-1/2,-y+1/2,-z+1
